# A disorder-related variant (E420K) of a PP2A-regulatory subunit (PPP2R5D) causes constitutively active AKT-mTOR signaling and uncoordinated cell growth

**DOI:** 10.1016/j.jbc.2021.100313

**Published:** 2021-01-20

**Authors:** Cinta M. Papke, Kali A. Smolen, Mark R. Swingle, Lauren Cressey, Richard A. Heng, Mourad Toporsian, Liyong Deng, Jacob Hagen, Yufeng Shen, Wendy K. Chung, Arminja N. Kettenbach, Richard E. Honkanen

**Affiliations:** 1 Department of Biochemistry and Molecular Biology, University of South Alabama, Mobile, Alabama, USA; 2 Department of Biochemistry and Cell Biology, Geisel School of Medicine at Dartmouth, Hanover, New Hampshire, USA; 3 Norris Cotton Cancer Center, Geisel School of Medicine at Dartmouth, Lebanon, New Hampshire, USA; 4 Department of Pediatrics, Columbia University Irving Medical Center, New York, New York, USA; 5 Department of Systems Biology, Columbia University Irving Medical Center, New York, New York, USA; 6 Herbert Irving Comprehensive Cancer Center, Columbia University Medical Center, New York, New York, USA; 7 Department of Medicine, Columbia University Medical Center, New York, New York, USA

**Keywords:** PPP2R5D, PP2A, phosphatase, PPP2R5D-intellectual disability, PPP2R5D-related neurodevelopmental disorder, Jordan’s syndrome, AKT-mTOR

## Abstract

Functional genomic approaches have facilitated the discovery of rare genetic disorders and improved efforts to decipher their underlying etiology. PPP2R5D-related disorder is an early childhood onset condition characterized by intellectual disability, hypotonia, autism-spectrum disorder, macrocephaly, and dysmorphic features. The disorder is caused by *de novo* single nucleotide changes in *PPP2R5D*, which generate heterozygous dominant missense variants. *PPP2R5D* is known to encode a B’-type (B’56δ) regulatory subunit of a PP2A-serine/threonine phosphatase. To help elucidate the molecular mechanisms altered in PPP2R5D-related disorder, we used a CRISPR-single-base editor to generate HEK-293 cells in which a single transition (c.1258G>A) was introduced into one allele, precisely recapitulating a clinically relevant E420K variant. Unbiased quantitative proteomic and phosphoproteomic analyses of endogenously expressed proteins revealed heterozygous-dominant changes in kinase/phosphatase signaling. These data combined with orthogonal validation studies revealed a previously unrecognized interaction of PPP2R5D with AKT in human cells, leading to constitutively active AKT-mTOR signaling, increased cell size, and uncoordinated cellular growth in E420K-variant cells. Rapamycin reduced cell size and dose-dependently reduced RPS6 phosphorylation in E420K-variant cells, suggesting that inhibition of mTOR1 can suppress both the observed RPS6 hyperphosphorylation and increased cell size. Together, our findings provide a deeper understanding of PPP2R5D and insight into how the E420K-variant alters signaling networks influenced by PPP2R5D. Our comprehensive approach, which combines precise genome editing, isobaric tandem mass tag labeling of peptides generated from endogenously expressed proteins, and concurrent liquid chromatography–mass spectrometry (LC-MS^3^), also provides a roadmap that can be used to rapidly explore the etiologies of additional genetic disorders.

PPP2R5D-related developmental disorder (OMIM#616355) is a syndrome characterized by moderate-to-severe developmental delay, intellectual disability, seizures, macrocephaly, autism-spectrum-disorder (ASD), hypotonia, and delayed motor skill development (1, 2, 3, 4). Genomic analyses revealed that the disorder is due to a *de novo* single-nucleotide missense mutation in the *PPP2R5D* gene (1, 2, 3, 5). To date, seven pathogenic variants of *PPP2R5D* have been reported (Fig. S1*A* and Table S1). All introduce a variant PPP2R5D protein harboring a single amino acid change in a highly conserved region. The molecular mechanisms that are affected by pathogenic PPP2R5D variants remain to be elucidated.

*PPP2R5D* encodes a B’-type subunit (B’56δ) of the phosphoprotein phosphatase type 2A (PP2A) holoenzyme. To date, upward of 72 PP2A-holoenzymes has been described. Nearly all are heterotrimeric protein complexes contain one of 18 unique B-type regulatory/targeting proteins and a ubiquitously expressed core dimer (Fig. 1*A*). The shared dimer is composed of a catalytic subunit (C-subunit) that functions as a serine/threonine protein phosphatase and a structural/scaffolding protein (A-subunit) that tethers the regulatory and catalytic subunits together. The B-subunits provide substrate specificity, affect intracellular localization, and regulate the catalytic activity of holoenzymes (6, 7, 8). In humans, two genes (*PPP2CA* and *PPP2CB*) encode nearly identical catalytic subunits and two genes (*PPP2R1A* and *PPP2R1B*) encode highly similar A-subunits. Each of the 18 known B-subunits is encoded by a distinct gene, with additional diversity generated by alternative splicing. After translation, the subunits do not simply coalesce with the core dimer to form a functional enzyme. Rather, holoenzyme biogenesis occurs through poorly characterized assembly and recycling mechanisms that pair a unique B-subunit with the common core dimer through organized and regulated processes (7, 9, 10).

**Figure 1:**
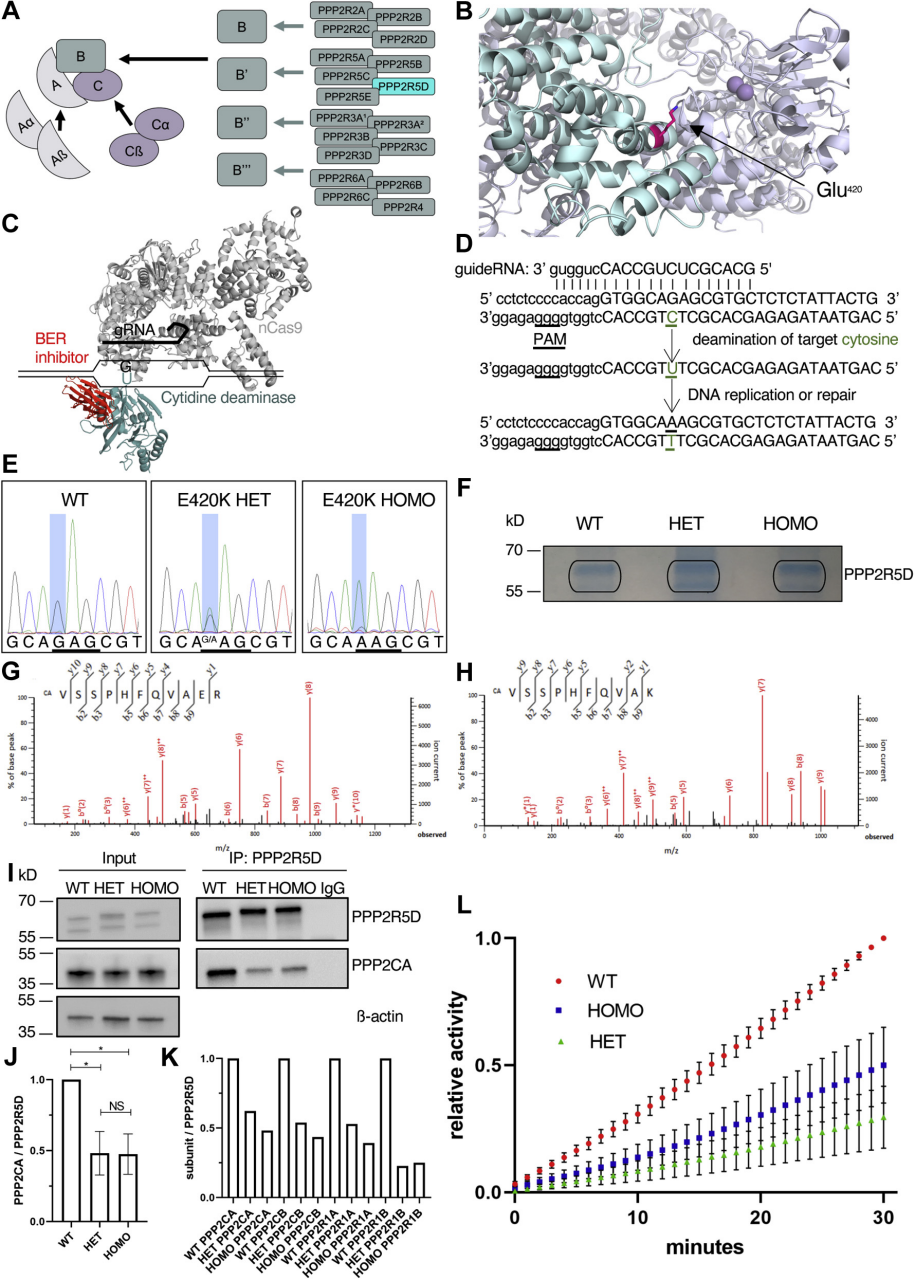
**Structure of PP2A holoenzymes and generation of PPP2R5D E420K-variant cell lines**. *A*, diagram of PP2A holoenzyme assembly with PPP2R5D in *cyan*. *B*, PPP2R5D homology model showing the likely location of the Glu^420^ variant in *pink* based on the crystal structure of PPP2R5C (PDB ID: 2IAE). PPP2R5D is colored as *cyan* and the scaffolding and catalytic subunits as *gray*. The catalytic metal ions are *purple*. *C*, illustration of targeted cytosine deamination by CRISPR/Cas9/BE4-Gam base editing. *D*, details of the gRNA interaction with the targeted genomic locus in exon 12 and genomic base editing. *E*, Sanger DNA sequencing data from WT encoding glutamic acid (GAG) in both alleles, E420K heterozygous (HET) encoding glutamic acid (GAG) in one allele and lysine (AAG) in the other, and E420K homozygous (HOMO) encoding lysine (AAG) in both alleles. *F*, IPs of endogenous PPP2R5D from WT, E420K HET, and E420K HOMO cells were separated by SDS-PAGE and stained with Coomassie Blue. LC-MS/MS spectra of peptides identified in E420K HET IP’s identified (*G*) wild type and (*H*) variant peptides. *I*, Western analysis of total cell extracts (input) and endogenous PPP2R5D IPs probed for PPP2R5D and PPP2CA. *J*, relative amounts of PPP2CA from WT or E420K cells normalized to total PPP2R5D in IPs and represented as the mean percent of WT (N = 5 independent experiments; mean ± SD, ANOVA Kruskal–Wallis *p* = 0.003 and H = 9.41, Dunn’s multiple comparison posthoc test, ∗*p* < 0.05 and NS: nonsignificant). *K*, LC-MS/MS detection of PPP2R1A, PPP2R1B, PPP2CA, and PPP2CB in PPP2R5D IPs of WT and E420K-variant cells. *L*, relative phosphatase activity of endogenous PPP2R5D IPs (n = 10). Endogenous PPP2R5D was immunoprecipitated from WT, HET, or HOMO cells and hydrolysis of DiFMUP was measured at Ex 360 nm and at Em 460 nm. Activity was normalized to total levels of PPP2R5D detected in IPs through immunoblotting shown in (*K*). WT activity was normalized at 30 min (endpoint) to 1 and variant activity is expressed relative to WT. N = 5 independent biological replicate of cells, each performed as n = 2 technical replicates.

Like many genetic developmental disorders, the etiology of PPP2R5D-related disorder is unknown. Human RNA-sequencing and protein expression data indicate that *PPP2R5D* is expressed in most tissues, with modestly higher levels in the brain, breast, testis, and gastrointestinal tissue (11, 12, 13). All PP2A-holoenzymes act as serine/threonine protein phosphatases, and most evidence for the involvement of “PP2A” in a particular process has been derived from the use of small-molecule inhibitors including okadaic acid (14), fostriecin (15, 16), and cantharidin (17, 18), which target the catalytic subunit shared among all PP2A-holoenzymes (19, 20), or siRNA, targeting a common core protein. Collectively, these studies have linked “PP2A” phosphatase activity to nearly all signaling networks controlled by reversible phosphorylation (21, 22, 23, 24). However, in a biological setting, individual holoenzymes are likely regulated independently, recognize limited specific substrates, and function in a limited number of intracellular processes (6, 7, 8). The challenge before the field is to decipher regulatory mechanisms and biological substrates of the >70 PP2A-holoenzymes.

To explore the biological role of PPP2R5D holoenzymes and decipher molecular mechanisms associated with a clinically relevant PPP2R5D variant, we used a CRISPR single base editor to generate HEK-293 cell lines in which a transition (c.1258G>A) introduced the E420K missense variant in *PPP2R5D*. The E420K variant was chosen for study because it is a reoccurring pathogenic variant (2), and the gene encoding PPP2R5D (*PPP2R5D*) contains an endogenous protospacer adjacent motif (PAM) ideally suited for single base editing of the target purine with BE4-Gam. Genomic editing allowed for cell-based studies of endogenously expressed proteins, and tandem mass tag (TMT) quantification and liquid chromatography coupled with mass spectrometry (LC-MS^3^) enabled unbiased quantitative global proteomic and phosphoproteomic analyses of wild-type (WT), E420K-heterozygous, and E420K-homozygous cells. Our studies reveal heterozygous dominant changes in kinase/phosphatase signaling in E420K variant cells, providing a foundation to help decipher the etiology of PPP2R5D-related developmental disorder.

## Results

### Generation of PPP2R5D E420K variant cell lines

To gain insight into how variants in *PPP2R5D* alter biological processes at a molecular level, we used BE4-Gam, a fourth-generation genomic base editor, to create stable HEK-293 cell lines containing a pathogenic E420K variant (Fig. 1*B*). To recapitulate the missense variant, we introduced a single base-pair change (c.1258G>A) in only one of the two alleles (Fig. 1*C*). Base editing was facilitated by a well-positioned endogenous PAM in *PPP2R5D* and the development of gRNA that positioned the cytidine deaminase precisely over the C:G target (Figs. 1*D* and S1*B*) while simultaneously positioning the nCas9 to nick the DNA strand containing the unedited guanine. Nicking the unedited DNA strand favors repair of the edited DNA using the uracil-containing strand as a template by endogenous mismatch repair mechanisms (25). The fusion of inhibitors of base excision repair (BER) helps prevent removal of the uracil generated by the deamination of cytidine, which increases efficiency of correct base editing (26). Cells were single-cell sorted 48 h after transfection to allow time for replication, after which the base edit becomes a permanent change in the genome.

By Sanger sequencing, we confirmed the genotype of cell lines containing the desired E420K heterozygous (HET) variant (Fig. 1*E*). We also developed cells in which the E420K variant was introduced into both alleles (HOMO). To ensure the cell lines were homogeneous, each clonal-derived line was single-cell sorted three times, prior to further analysis (Fig. S1*C*). Complete genomic sequencing of the parental, E420K HET, and E420K HOMO variant cell lines was performed to characterize the genomic background, detect any off-target editing, and ensure that the repetitive single-cell sorting did not introduce spontaneous somatic mutations. Cell lines with off-target or spontaneous mutations in protein-coding regions were discarded.

### The PPP2R5D E420K variant is expressed

Having established that the cell lines were homogeneous and contained the desired zygosity of the *PPP2R5D* c.1258G>A variant allele, we examined the expression and assembly of the endogenously expressed variant protein into a holoenzyme. Immunoprecipitates (IPs) of endogenous PPP2R5D were Coomassie-blue stained (Fig. 1*F*) and analyzed by LC-MS/MS revealing peptides containing Glu^420^ or Lys^420^ in the HET extracts (Fig. 1, *G* and *H*). As expected, only the Glu^420^ peptide was identified in extracts generated from WT, and only the Lys^420^ peptide was identified in HOMO extracts. Detection of C-terminal peptides indicates that the E420K mutation does not compromise the production of full-length PPP2R5D protein. Orthogonal validation of the LC-MS/MS data was obtained through western analysis, which revealed similar levels of PPP2R5D in both total cell extracts and IPs generated from all three cell lines (Fig. 1*I*). Proteins that participate in PP2A biogenesis, including LCMT1, PME1, alpha4, TIPRL, and PTPA, were similarly expressed in all three cell lines (Table S2). Western and LC-MS/MS analysis revealed the presence of both catalytic (Fig. 1, *I–K*) and scaffolding (Fig. 1*K*) subunits in PPP2R5D IPs of WT and E420K variant cells. This observation indicates that the E420K variant does not prevent the biogenesis of holoenzymes containing PPP2R5D. Our studies also revealed that both WT and E420K variant PPP2R5D assemble with either isoform of the catalytic (PPP2CA/PPP2CB) or scaffold (PPP2R1A/PPP2R1B) subunits, suggesting that PPP2R5D can be incorporated into four distinct holoenzymes. It should be noted that reduced levels of PPP2CA/B and PPP2R1A/B were found in PPP2R5D IPs of E420K variant cells, suggesting the partial loss of PPP2R5D-holoenzyme formation or decreased stability.

We next tested the E420K variant containing holoenzymes for phosphatase activity using an established fluorescent (DiFMUP) assay (27, 28, 29, 30). In this assay, PPP2R5D was immunoprecipitated using an antibody that recognizes a near C-terminal epitope that is unique to PPP2R5D, and the phosphatase activity of the associated PP2A-holoenzyme was measured in the IP (Fig. 1*L*). To adjust for minor technical variation inherent in IP-based assays, the activity associated with WT and variant immunoprecipitated PPP2R5D-phosphatase complexes was normalized to total levels of PPP2R5D detected in the IPs by immunoblotting analyses. Currently, this approach represents the only viable method to measure the activity of endogenously expressed PPP2R5D-harboring holoenzymes without contaminating activity from one of the other B’-family members that share considerable homology (PPP2R5A, PPP2R5B, PPP2R5C, and PPP2R5E) (Fig. S2*A* and Table S3). We observed the dephosphorylation of DiFMUP in all samples, with less activity in the IPs generated from E420K variant cells, which is consistent with observed reduced levels of immunoprecipitated catalytic subunit. When the data was normalized to the amount of catalytic subunit in the IP, the activity was similar in WT and E420K variants (Fig. S2*B*), suggesting that the specific activity of the variant holoenzymes was not reduced. From these studies, we concluded that the E420K variant is expressed and translated. Future studies will be needed to determine if the reduced catalytic activity in the E420K variants reflects partial assembly, altered recycling, or decreased stability of PP2A-variant holoenzymes.

### Quantitative changes in the proteome and phosphoproteome in PPP2R5D E420K variant cells

**Figure 2:**
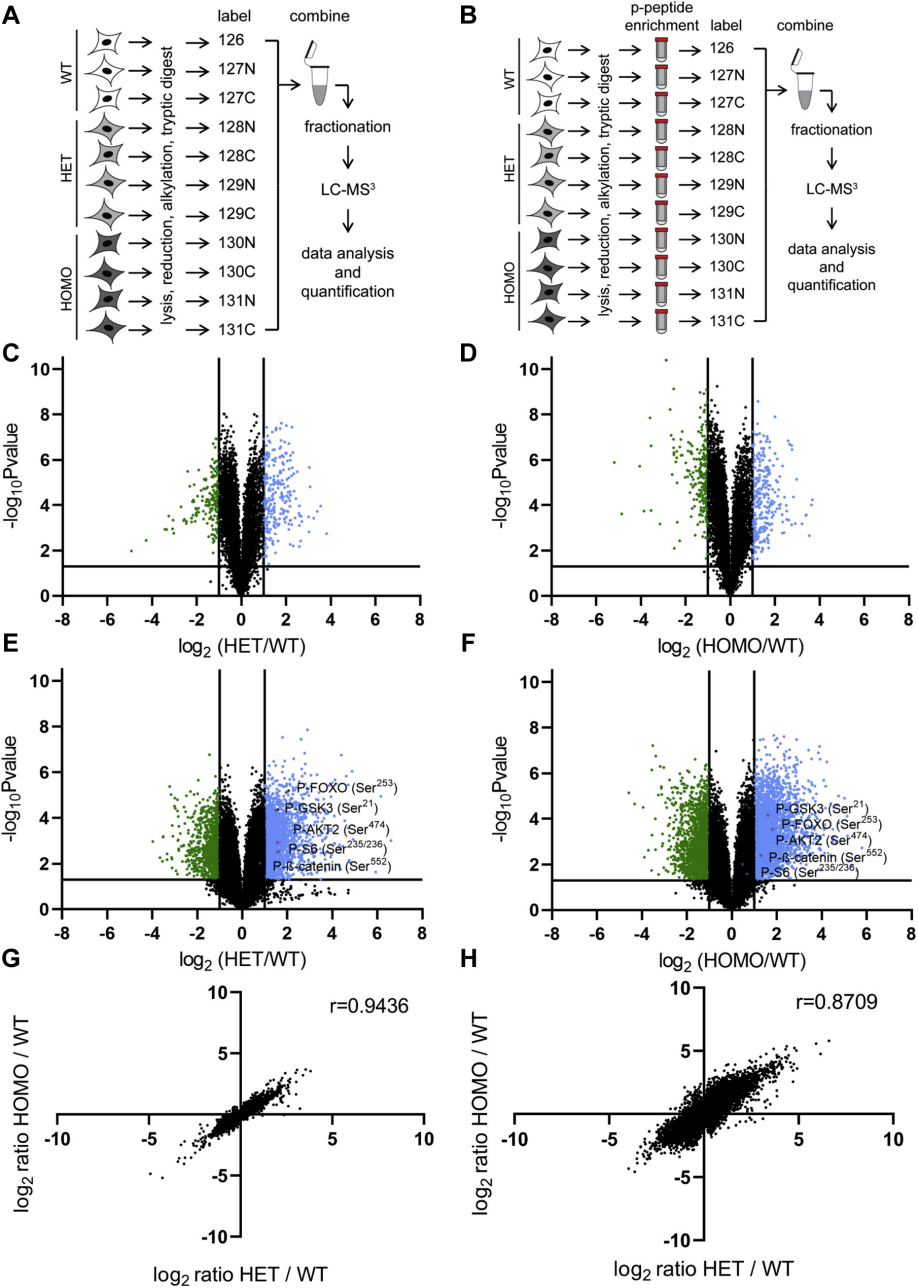
**Quantitative changes in the proteome and phosphoproteome in PPP2R5D E420K-variant cells.** Workflow for quantitative proteomics (*A*) and phosphoproteomics (*B*). Volcano plots of proteomic (*C*, *D*) and protein-corrected phosphoproteomic (*E*, *F*) data (N = 3 independent biological replicates for WT, N = 4 independent biological replicates for E420K HET and HOMO). Volcano plots depict log_2_ ratios of peptides, plotted against the negative log_10_ of the *p* value of their fold change. Peptides above –log_10_ = 1.3 (corresponding to *p* < 0.05) are considered statistically significant. Peptides shown in green or blue are twofold or more decreased or increased in abundance, respectively. Correlation analysis of proteomic (*G*) and phosphoproteomic (*H*) between E420K HET and HOMO datasets.

A major hurdle to determining how the E420K mutation alters the biological functions of PPP2R5D is that only a few studies have explored the “normal” action(s) of PPP2R5D at a cellular level (31, 32). Therefore, we chose a global unbiased approach, employing TMT labeling for relative and absolute quantitation with LC-MS^3^, to obtain proteomic (Fig. 2*A*) and phosphoproteomic (Fig. 2*B*) data sets comparing WT and E420K variant cells. Overall, we reproducibly quantified 26,731 phosphopeptides on 5926 proteins in the phosphoproteomic analysis and 9178 proteins in the proteomic analysis (Table S2 and Fig. S2*C*). Most proteins did not change in abundance (Fig. 2, *C* and *D*). Only 2.6% of all proteins quantified were significantly increased or decreased by twofold or more in E420K HET compared with WT cells. Notably, large changes in the abundance of PP2A catalytic, scaffolding, regulatory, or other PPP-subunits were not detected. However, approximately twofold changes in some PP1R-subunits were observed (Table S4). In contrast, 8.8% of phosphopeptides were significantly increased or decreased by twofold or more in E420K HET compared with WT cells (Table S2) suggesting that the E420K variant alters global protein phosphorylation, which is consistent with previous reports indicating that PPP2R5D acts as a regulator of PP2A-holoenzyme function (6, 13, 33, 34, 35, 36). To help distinguish changes in phosphopeptide abundance resulting from changes in protein levels *versus* changes in protein phosphorylation, we further filtered the data set, correcting for changes in protein abundances. This reduced our data set to 24,307 reproducibly identified and quantified phosphopeptides (Fig. 2, *E* and *F*), of which 7% were significantly increased or decreased in E420K HET compared with WT cells. Compared with WT, E420K HET cells had more phosphopeptides increasing phosphorylation occupancy than phosphopeptides decreasing phosphorylation occupancy (1.9-fold). Correlation analyses of the E420K HOMO and HET proteomics and phosphoproteomics data sets revealed a strong positive correlation for both HET and HOMO samples (Fig. 2, *G* and *H*) indicating that the data sets were highly similar. This observation is consistent with the heterozygous variant acting as a dominant condition in humans.

### Enrichment of R-x-R-x-x-S/T-B phosphopeptide motif in PPP2R5D E420K variant cells

**Figure 3:**
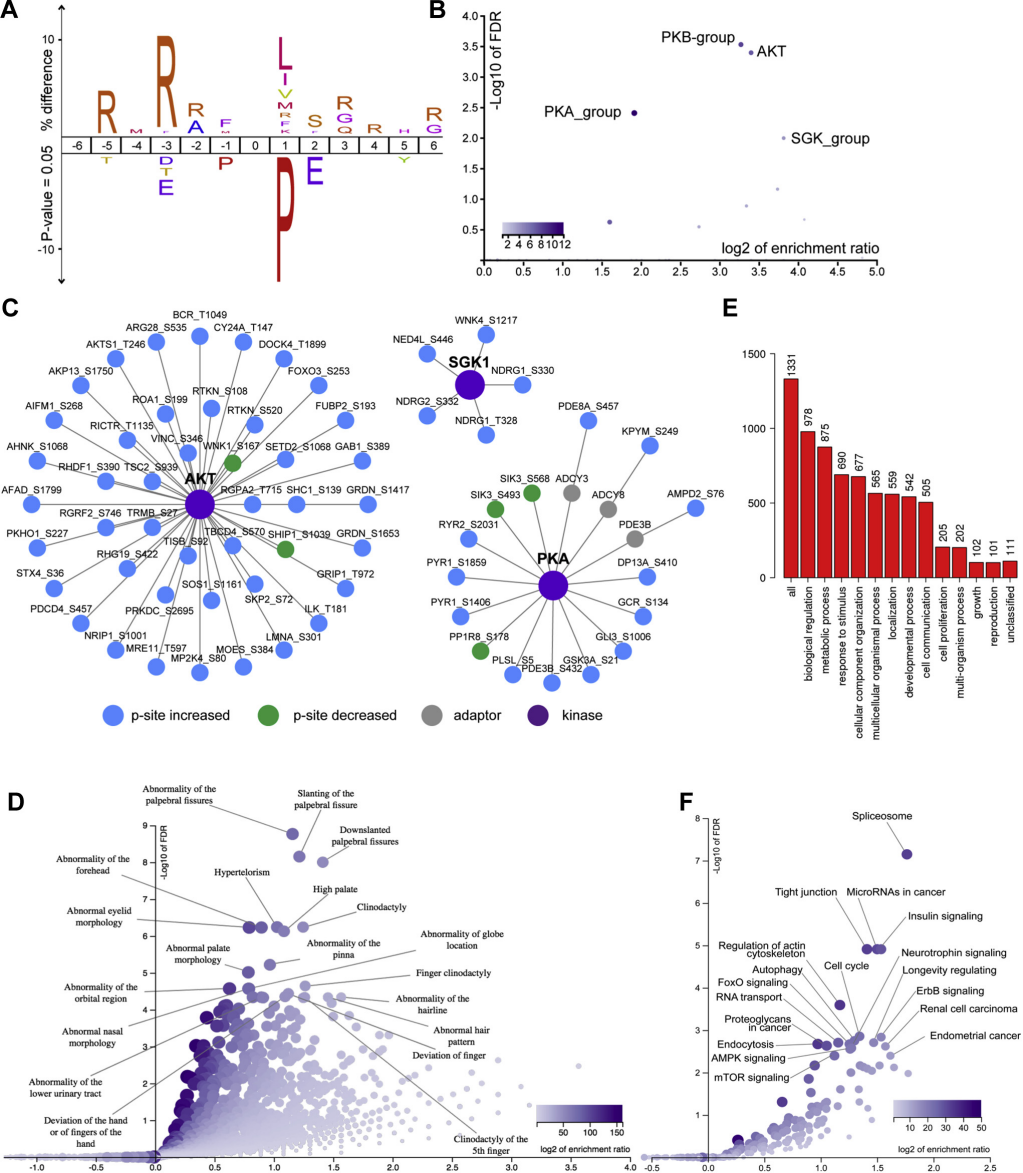
**Peptide phosphorylation and phenotype analysis of proteins with altered phosphorylation site occupancy in PPP2R5D E420K-variant cells.***A*, Icelogo displaying enrichment and deselection of amino acids surrounding the phosphorylated residue (position 0) and (*B*) RegPhos analysis of kinase substrates enriched in significantly regulated, localized, single phosphorylation sites compared with all localized, single phosphorylation sites identified in the analysis. *C*, significantly regulated, localized, single phosphorylation sites in E420K-variant cells known or predicted to be phosphorylated by the indicated kinase. Node color (*blue* increased, *green* decreased, *yellow* both increased and decreased) indicates type of phosphorylation site regulation. Edges indicate known or predicted upstream kinase. *D*, overrepresentation analysis of human phenotype ontology data base. *E*, Gene ontology analysis of enriched biological processes. *F*, overrepresentation analysis of human KEGG pathways data.

To identify signaling cascades altered in the variant cell lines, we investigated the possibility that specific kinases might differentially oppose PPP2R5D WT and E420K-variant cells. Overrepresentation analysis identifying consensus sequences either enriched or deselected, revealing an enrichment of an R-x-R-x-x-S/T-B motif (Fig. 3*A*) where x represents any amino acid and B represents amino acids with hydrophobic side chains. This motif is a protein kinase B (AKT/PKB) consensus sequence, which is shared by PKCα, PKCß, and RSK1 (37). We also observed a strong deselection of proline-directed motifs in phosphorylation sites differentially regulated in the E420K variant compared with WT cell lines (Fig. 3*A*). These observations are consistent with both the phosphorylation site consensus motif preferences and the phosphorylation site consensus motif disfavors we observed for the PPP2R5-holoenzymes families (31). Performing the enrichment analysis independently for phosphorylation sites that were significantly changed revealed that the enrichment of the AKT consensus motif was due to sites increased in phosphorylation (Fig. S3*A*). The hyperphosphorylation of AKT, PKC, and RSK1 substrates has been previously reported following treatment with okadaic acid (38), which is often reported as a specific inhibitor of PPP2CA but actually also inhibits PPP4C, PPP5C, and PPP6C with similar potency (19, 20, 39, 40, 41).

Bioinformatics tools (KinomeXplorer; PhosphoSitePlus; RegPhos) were used to predict kinases responsible for the observed changes in phosphorylation and to identify kinase–substrate regulatory networks, revealing enrichment of AKT, serum and glucocorticoid-regulated kinase (SGK), and cAMP-dependent protein kinase (PKA) substrates among the phosphorylation sites increased in E420K-variant cells (Fig. 3*B* and Table S5). Indeed, most known and predicted AKT, SGK1, and PKA phosphorylation sites showed increased phosphorylation in variant cell lines (Fig. 3*C* and Table S5). Together, these data are consistent with the E420K variant having a dominant loss of suppression effect on AKT-mediated signaling cascades.

For interrogation of the phenotype ontology database, we filtered the number of phosphopeptides in the protein-corrected data set to those that were significantly increased or decreased by twofold in the variant *versus* WT group. We used the Human Phenotype Ontology database to identify enriched phenotypes (Fig. 3*D*). Although these experiments were conducted in HEK-293 cells, a diverse array of phenotypes were enriched in our analysis, including many observed in children diagnosed with PPP2R5D-variant disorder (4). Due to the prevalence of neurodevelopmental disorders (NDD) in PPP2R5D-variant affected children, we also investigated which genes corresponding to proteins with significantly altered phosphorylation sites are associated explicitly with NDD. Overenrichment analysis identified 262 unique entrez gene IDs that were annotated to the selected functional categories in common with the reference list (Table S6). Of the top 20 identified genes, 11 were genes associated with NDD curated by four sources: SFARI gene score 1/2; ASC FDR <0.1 DDG2P (DDD curated) genes; DDD FDR <0.025 (42, 43, 44) (Table S7). There are a total of 627 genes in the curated NDD gene list. Given a total of >19,000 protein-coding genes, 11/20 overlap with a 627-gene list gave us a *p*-value of 1e-11, with an odds ratio of 36. This is a conservative estimate, given how many NDD genes are putative targets of PPP2R5D. These data are consistent with clinical presentations in PPP2R5D patients including neurodevelopmental delay and macrocephaly, a phenotype associated with increased AKT activity.

### Metabolic AKT-mTOR signaling is altered by the PPP2R5D E420K variant

**Figure 4:**
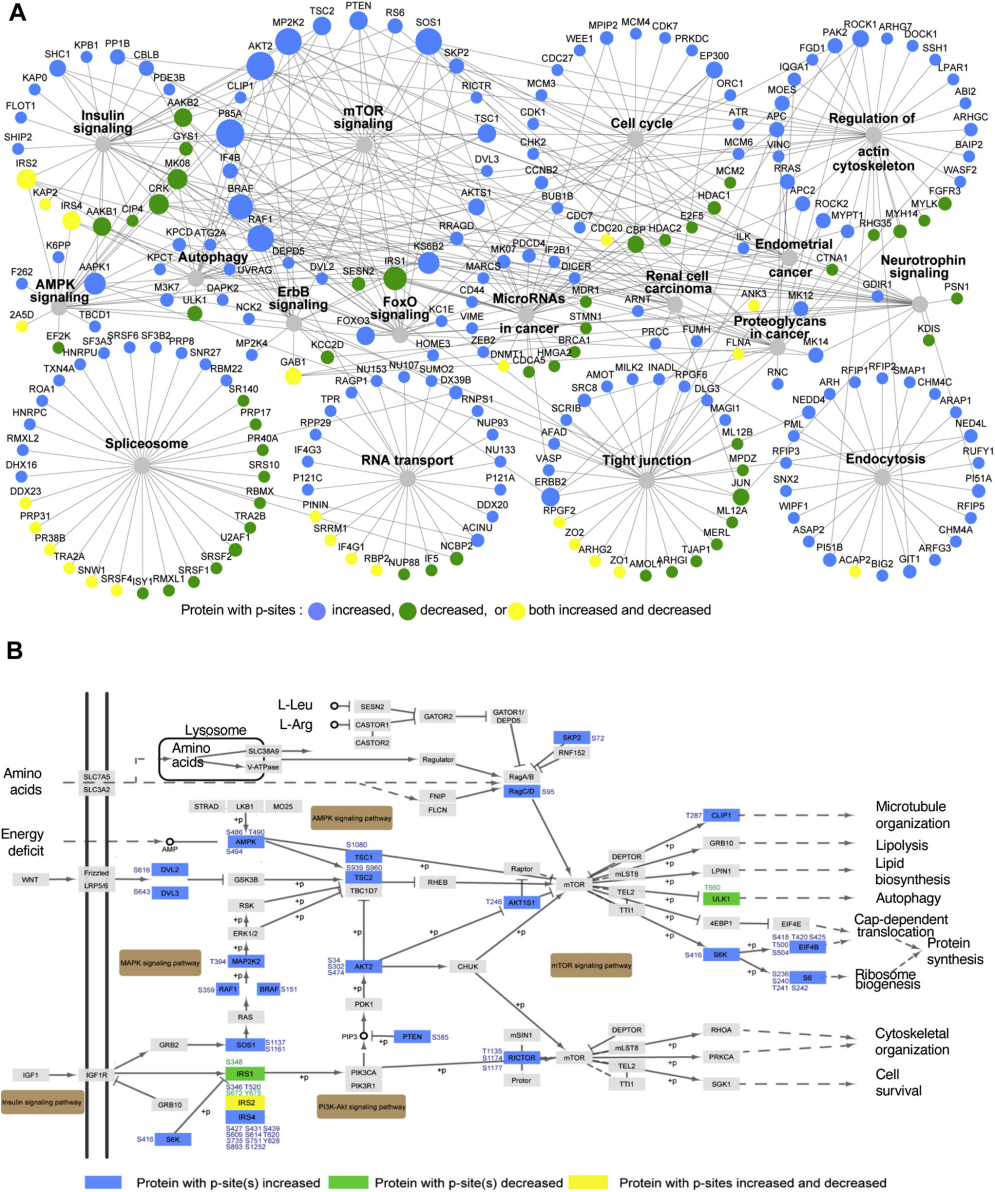
**Bioinformatic pathway analysis of proteins with altered phosphorylation site occupancy in PPP2R5D E420K-variant cells.***A*, proteins associated with enriched KEGG pathways identified in Figure 3*F*. Node size correlates with the number of pathways the protein is associated with. Node color indicates the type of phosphorylation site regulation. Edges indicate pathway association. *B*, detailed diagram of enriched KEGG pathways. Proteins with p-site(s) increased are shown in *blue*, proteins with p-site(s) decreased are *green*, and proteins with p-sites both increase and decrease are *yellow*.

Studies with inhibitors of the common PP2A catalytic subunit have linked “PP2A activity” (representing the combined activity of >70 PP2A-holoenzymes) to the regulation of virtually every signaling cascade influenced by serine/threonine kinases. To gain insight into processes specifically altered by the PPP2R5D E420K variant, the same filtered data set used for phenotype ontology resulted in the identification of 2457 phosphorylation sites on 1382 proteins, of which 763 and 1694 were significantly decreased or increased, respectively. This data set revealed several differences in metabolic signaling pathways between WT and E420K-variant cells (Fig. 3*E* and Table S6). Specifically, we identified hyperphosphorylated proteins in insulin, mTOR, AMPK, FoxO, and ErbB signaling, along with downstream effectors such as 40S ribosomal protein S6 (RPS6), eukaryotic translation initiation factor 4E-binding protein 1 (4EBP1), and eukaryotic translation initiation factor 4B (EIF4B) that control cell size and protein synthesis (Figs. 3*F* and 4*A*; Fig. S3, *B–E* and Table S6). We identified three hyperphosphorylated sites (Thr^1135^, Ser^1174^, Ser^1177^) on the rapamycin-insensitive companion of mTOR (RICTOR), which is part of the mTOR2 complex that phosphorylates AKT at Ser^473/4/2^ (Fig. 4*B*). AKT (isoform 2 based on phosphopeptide sequence) was hyperphosphorylated at several sites (Ser^34^, Ser^302^, Ser^474^) (Fig. 4*B*). P-Ser^473^ (Ser^474^ on isoform 2) increases the kinase activity of AKT (45). Downstream of AKT, the negative regulator of mTOR1, AKT1S1/PRAS40, was hyperphosphorylated at Thr^246^. P-Thr^246^ is known to relieve AKT1S1/PRAS40 inhibitory functions toward mTOR1 (46), resulting in mTOR1 activation. Another negative regulator of mTOR1, tuberin (TSC2), was hyperphosphorylated at Ser^939^ and Ser^960^. AKT phosphorylates TSC2 at Ser^939^ to relieve TSC2-directed inhibition of mTOR1 (47). Downstream effectors of mTOR1, such as 40S ribosomal protein S6-Kinase (S6K) (Ser^416^), were also hyperphosphorylated. RPS6 can be phosphorylated by S6K and was found to be hyperphosphorylated at multiple sites (Ser^236^, Ser^240^, Thr^241^, and Ser^242^) in the E420K-variant cell lines. Another well-established substrate of AKT, glycogen synthase kinase 3α (GSK3α) (48), was hyperphosphorylated at Ser^21^ in E420K-variant cells suggesting inhibited GSK3α activity. Together these phosphoproteomic data indicate that PPP2R5D E420K variants act as dominant constitutive activators of AKT-mTOR signaling.

### Orthogonal validation of the LC-MS^3^ data

**Figure 5:**
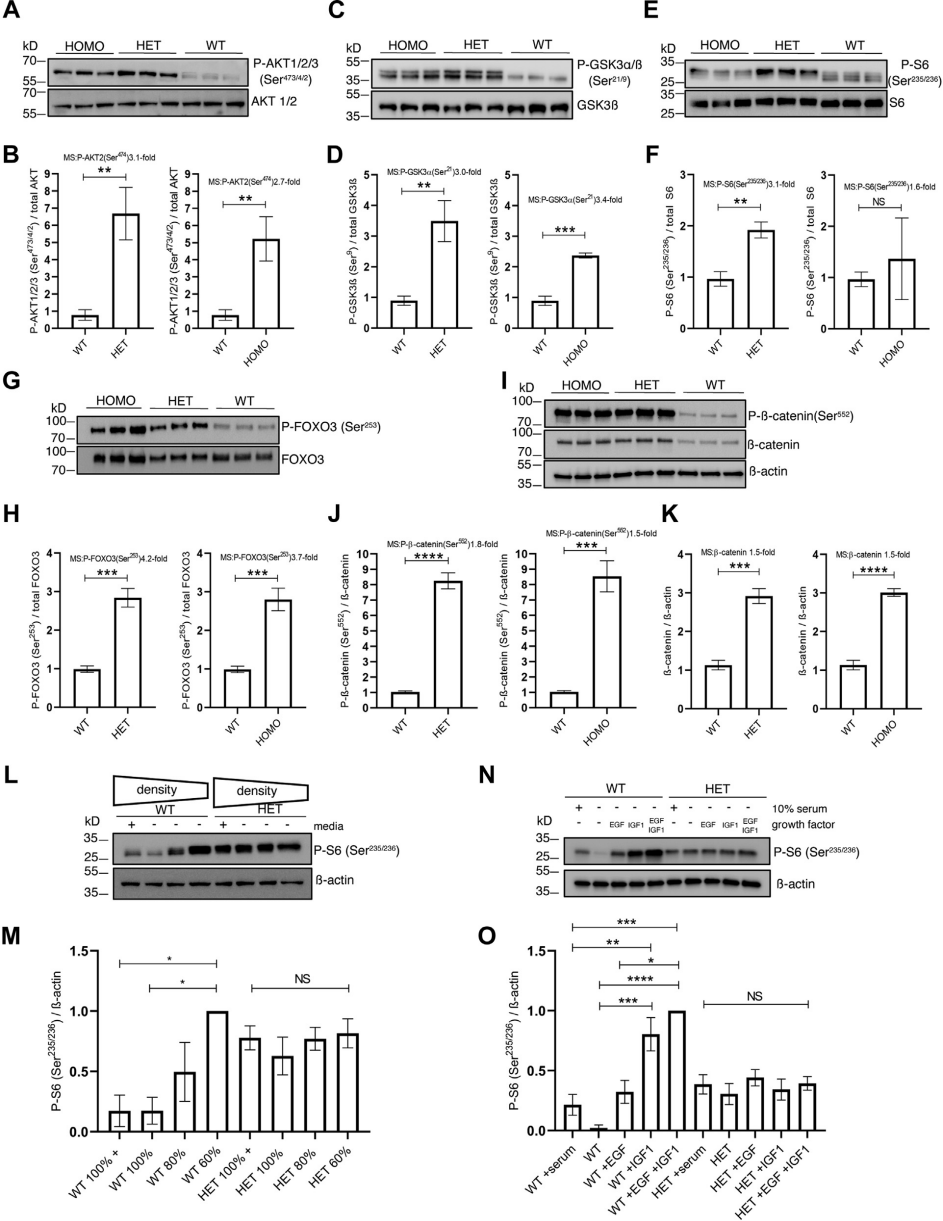
**Orthogonal validation of the LC-MS**^**3**^**data.***A*, representative immunoblot and (*B*) associated graph showing the levels of P-AKT1/2/3 (Ser^473/4/2^) normalized to total AKT1/2. (*C*) Representative immunoblot and (*D*) associated graph showing the levels of P-GSK3α/ß (Ser^21/9^) normalized to total GSK3ß. *E*, representative immunoblot and (*F*) associated graph showing the levels of P-S6 (Ser^235/236^) normalized to total S6. *G*, representative immunoblot and (*H*) associated graph showing the levels of P-FOXO3 (Ser^253^) normalized to total FOXO3. *I*, representative immunoblot and (*J*) associated graph showing the levels of P-ß-catenin (Ser^552^) normalized to total ß-catenin and (*K*) associated graph showing levels of total ß-catenin normalized to ß-actin. All (*A–K*) were performed as n = 3 independent cell experiments, mean ± SD, unpaired *t* test ∗∗*p* < 0.01 ∗∗∗*p* < 0.001, ∗∗∗∗*p* < 0.0001. MS values indicate the fold change detected in the LC-MS dataset (Fig. S2). *L*, representative immunoblot and (*M*) associated graph showing levels of P-S6 (Ser^235/236^) normalized to total ß-actin. Cells were assayed in 10% serum containing media at decreasing cell densities. “+” sign indicates 100% confluent cells incubated with fresh media containing 10% serum for 24 h prior to harvest. (n = 3 independent experiments, mean ± SD, ANOVA Kruskal–Wallis *p* = 0.008 and H = 19.11, Dunn’s multiple comparison posthoc test, ∗*p* < 0.05 and NS: nonsignificant). *N*, representative immunoblot and (*O*) associated graph showing levels of P-S6 (Ser^235/236^) normalized to total ß-actin. Cells were grown in the presence/absence of 10% serum for 24 h and stimulated with/without EGF (0.5 ng/ml) and/or IGF-1 (0.5 ng/ml) for 30 min prior to harvest. (n = 4 independent experiments, mean ± SD, ANOVA Kruskal–Wallis *p* < 0.0001 and H = 45.3, Dunn’s multiple comparison posthoc test, ∗*p* < 0.05, ∗∗*p* < 0.01, ∗∗∗*p* < 0.001, ∗∗∗∗*p* < 0.0001, and NS: nonsignificant).

Quantitative LC-MS^3^ revealed 3.1-fold hyperphosphorylation of AKT2 on Ser^474^ in E420K HET compared with WT cells. The epitope surrounding Ser^474^ is conserved in AKT1, AKT2, and AKT3 (Ser^473^, Ser^474^, and Ser^472^, respectively), and a phospho-specific antibody recognizes all three phosphorylated AKT isoforms. Consistent with the LC-MS^3^ data, western analysis revealed increased phosphorylation in the E420K HET cells (Fig. 5, *A* and *B*). Ser^474^ phosphorylation activates AKT activity, resulting in GSK3α (Ser^21^) and GSK3β (Ser^9^) phosphorylation and inhibition of GSK3 activity (48). The epitopes surrounding Ser^21^ and Ser^9^ are identical and recognized by a single phospho-specific antibody. Western analysis revealed increased levels of P-GSK3α/ß (Ser^21/9^) in E420K-variant cell extracts (Fig. 5, *C* and *D*), consistent with our phosphoproteomic analysis showing a 3.0-fold increase in P-GSK3α in E420K HET cells compared with WT. LC-MS^3^ analysis of variant cells revealed that RPS6 phosphorylation on Ser^235^, Ser^236^, and doubly Ser^235/236^ was elevated by 2.2-fold, 5.3-fold, and 3.1-fold, respectively in E420K HET compared with WT extracts. A triple phosphorylation of RPS6 with further phosphorylation on Ser^240^ or Thr^241^ was also increased in the E420K HET, but not the E420K HOMO variants (Table S8). Western analysis assessing doubly phosphorylated RPS6 at Ser^235/236^ revealed increased phosphorylation in E420K HET extracts (Fig. 5, *E* and *F*). Western analysis of other known AKT targets also revealed increased phosphorylation in variant cells, including P-FOXO3 (Ser^253^) (Fig. 5, *G* and *H*) and P-ß-catenin (Ser^552^) (Fig. 5, *I* and *J*). Increased ß-catenin protein levels (Fig. 5*K* and Table S2) were associated with decreased P-ß-catenin (Ser^33/37^) (Fig. S4, *A* and *B*). Similar results were observed in the E420K homozygous cell lines (Fig. 5, *A*–*K*). When considered in their entirety, these observations are most consistent with dominant aberrant AKT-mTOR signaling induced by the PPP2R5D E420K variant.

### PPP2R5D E420K variant cells have a constitutively active pathway involving RPS6

For western and LC-MS^3^ analyses, cell extracts were generated from nearly confluent monolayers and yielded consistent differences in RPS6 phosphorylation (Ser^235/236^). However, phosphorylation of RPS6 can be influenced by cell density (49) and growth factors (50). Therefore, changes in RPS6 phosphorylation required more rigorous analysis. We observed elevated phosphorylation of RPS6 (Ser^235/236^) in WT cells growing at low density, which decreased with increasing cell density (Fig. 5, *L* and *M* and Fig. S4*C*). In contrast, E420K HET cells growing at low density displayed elevated baseline phosphorylation of RPS6 that did not change with increasing cell density (Fig. 5, *L* and *M* and Fig. S4*C*). Serum deprivation is known to abolish RPS6 Ser^235/236^ phosphorylation and was observed in WT cells, but not in HET cells (Fig. 5, *N* and *O*). The addition of epidermal growth factor (EGF), insulin-like growth factor 1 (IGF1), or a combination of both under serum-free conditions increased P-RPS6 above the level observed in the serum-supplemented high-density WT cells. In contrast, baseline P-RPS6 was elevated to a submaximal level in serum-deprived HET cells, and subsequent treatment with either EGF, IGF, or a combination of both did not elicit an increase in P-RPS6 above basal levels. Additionally, LC-MS^3^ analyses revealed 1.3-fold decrease in IGF1 receptor (IGF1R) protein levels in HET cells compared with WT cells, which may reflect feedback inhibition of IGF1 signaling due to the constitutive activation of AKT in this pathway (Table S2).

### Rapamycin inhibits aberrant mTOR signaling and decreases cell size in PPP2R5D E420K variant cells

**Figure 6:**
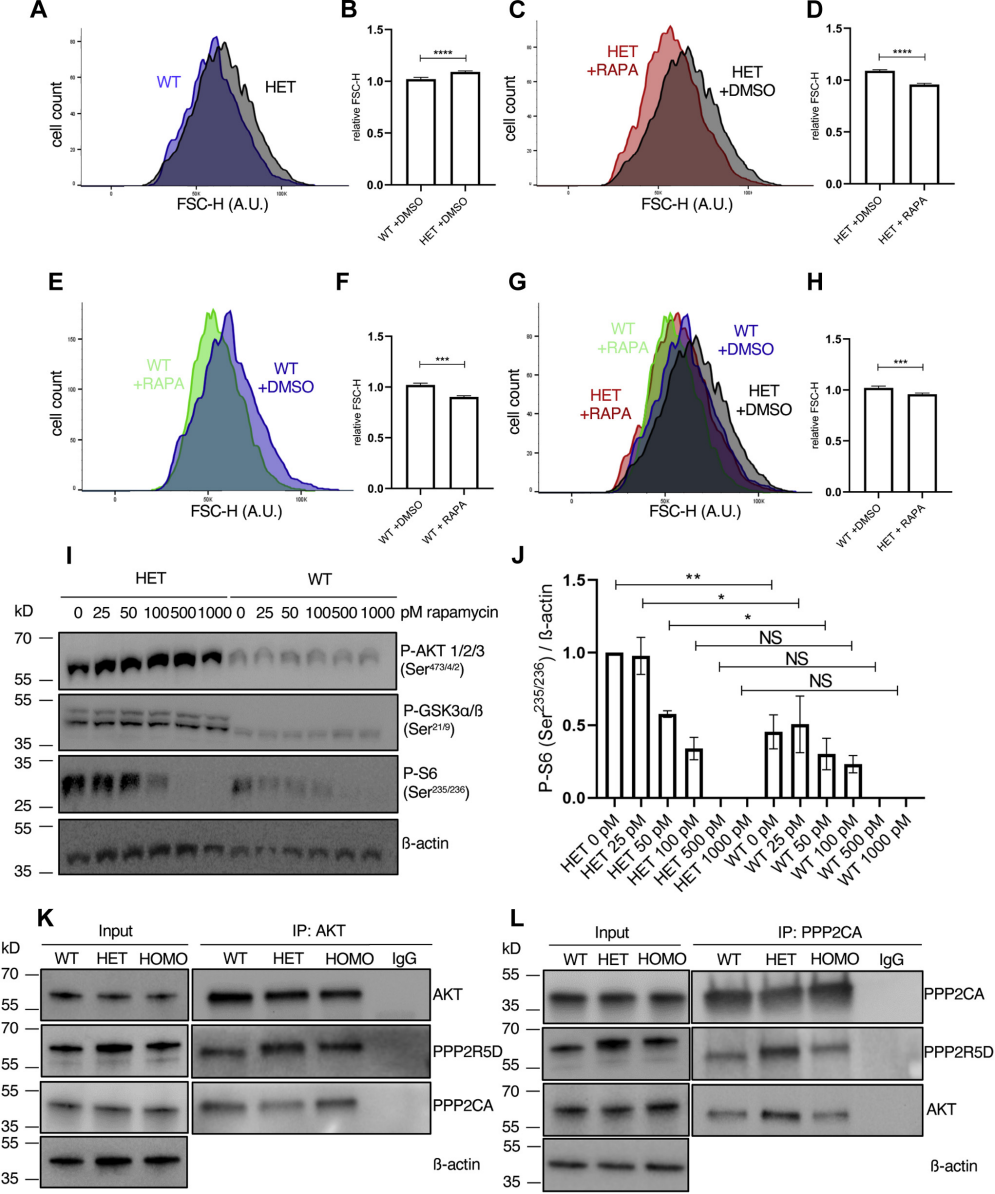
**Rapamycin inhibits aberrant mTOR signaling and decreases cell size in E420K-variant cells.***A*, representative histogram showing the distribution of mean FSC-H for HET cells (*black*) compared with WT cells (*blue*). *B*, graph showing greater mean relative FSC-H in HET compared with WT cells. Treatment with rapamycin (10 nM, 48 h) significantly decreased the mean relative cell sizes of both HET (*C*, *D*) and WT (*E*, *F*) cells. The absolute rapamycin-induced decrease in HET (*red*) was beyond that seen in WT DMSO-control treated cells (*blue*) *G*, *H*, FSC-H is in arbitrary units (AU) relative to each other (n = 4 independent experiments; mean ± SD, unpaired *t* test ∗∗∗*p* < 0.001, and ∗∗∗∗*p* < 0.0001). *I*, representative immunoblot showing dose-dependent inhibition of P-S6 (Ser^235/236^) by rapamycin. Rapamycin did not affect P- AKT1/2/3 (Ser^473/4/2^) and P-GSK3α/ß (Ser^21/9^). ß-actin was probed as an internal control. *J*, graph showing levels of P-S6 (Ser^235/236^) normalized to ß-actin (n = 3 independent experiment, shown is the representative experiment). *K*, representative immunoblot analysis of AKT IPs probing for PPP2CA and PPP2R5D. *L*, representative immunoblot analysis of PPP2CA IPs probing for PPP2R5D and AKT.

mTOR signaling regulates cell size through phosphorylation-dependent activation of RPS6 (51). A comparison of the relative cell size, determined by measuring mean forward scattering height (FSC-H) (51), revealed that E420K HET cells were significantly larger, evidenced by a rightward shift in FSC-H and a mean increase in FSC-H (Fig. 6, *A* and *B*). Treatment with rapamycin is known to decrease cell size by inhibiting mTOR1 signaling (51). We observed that rapamycin decreased FSC-H in both WT cells and E420K HET (Fig. 6, *C*–*F*). After 48 h, rapamycin-treated E420K and WT cells were similar in size, both smaller than respective solvent-treated controls (Fig. 6, *G* and *H*). Rapamycin also reduced P-RPS6 in both WT and HET cells in a dose-dependent manner (Fig. 6, *I* and *J*) suggesting that both the observed RPS6 hyperphosphorylation and increased cell size can be suppressed through the inhibition of mTOR1. In contrast, the elevated P-AKT and P-GSK3, which act upstream of the target of rapamycin, mTOR1, were not suppressed by rapamycin in the variant cells (Fig. 6*I*). This suggested that the PPP2R5D E420K variant affects signaling upstream of mTOR1.

### PPP2R5D-PPP2CA forms a complex with AKT

**Figure 7:**
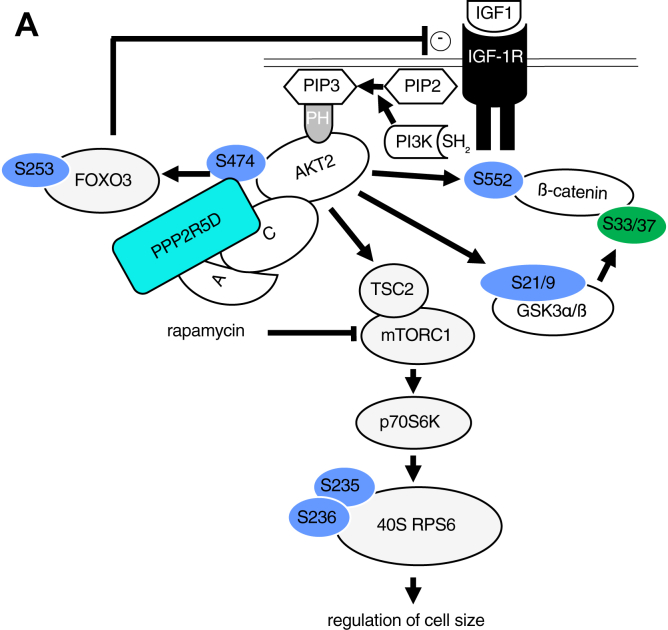
**PPP2R5D E420K-variant signaling.** Diagram of pathway regulation by PPP2R5D containing PP2A-holoenzymes altered in E420K variant HEK-293 cells based on the data presented. Phosphorylation sites shown in *blue* (hyperphosphorylated) and *green* (hypophosphorylated) differ in the E420K variant cells as compared with wild-type cells.

Our data are consistent with a PPP2R5D-holoenzyme directly opposing AKT, with the E420K variant cells having reduced the ability to inactivate AKT due to the failure to dephosphorylate Ser^473/4/2^. This data was further validated by IP-western analysis, in which both endogenous PPP2CA and PPP2R5D co-IP with AKT and both PPP2R5D and AKT co-IP with endogenous PPP2CA in WT and E420K cell extracts (Fig. 6, *K* and *L* and Fig. S5, *A–C*). Together, the data supports the existence of a protein complex containing AKT and a PPP2R5D-containing PP2A holoenzyme, with the E420K variant having a dominant loss of suppression effect on AKT-mediated signaling cascades (Fig. 7*A*).

## Discussion

Collectively PP2A-phosphatases represent up to 0.2% (52) of total cellular protein, and studies with catalytic inhibitors (*i.e.*, okadaic acid, fostriecin, or cantharidin) or siRNA (targeting subunits of the common core dimer protein) indicate that, as a family, PP2A phosphatases are important for the vast majority of cellular processes (53, 54, 55, 56, 57, 58, 59, 60). However, little is known about the biological actions of individual holoenzymes, and nothing has been reported related to how pathogenic PPP2R5D variants alter function. Here, employing single base genomic editing, we created human cell lines that recapitulate a clinically relevant pathogenic variant. The advantage of genomic editing is that the expression, translation, and biogenesis of the E420K variant holoenzyme are controlled by endogenous mechanisms. This is important because PP2A-holoenzyme biogenesis requires the coordinated actions of many proteins (*i.e.*, IGBP1/alpha4, PPP2R4/PTPA; LCMT1; PPPME1, and TIPRL) that are also needed for the biogenesis of the other 10 to 72 PP2A holoenzymes found in most cells (6, 7). Biogenesis starts with the synthesis of an inactive catalytic subunit (C-subunit) that undergoes a number of conformational changes and modifications before it gains catalytic activity and is incorporated into the common A/C core dimer. Functional PP2A-holoenzymes are created when a unique B-subunit is incorporated into the common core dimer (6, 7). Our studies revealed that both the WT and the E420K variant are expressed at similar levels. Our findings further our understanding of WT PPP2R5D and E420K-variant function, revealing its assembly with both isoforms of the catalytic (PPP2CA, PPP2CB) and scaffolding (PPP2R1A, PPP2R1B) subunits. This suggests that the E420K variant does not prevent biogenesis. However, when PPP2R5D is immunoprecipitated, IPs of E420K variant cells contained reduced levels of the catalytic subunit (PPP2CA/PPP2CB). This suggests that there may be a pool of unbound PPP2R5D in the cells. Alternatively, the variant containing holoenzymes may be less stable or some aspect of the holoenzyme biogenesis/recycling process may be altered, which warrants further investigation in future studies of PPP2R5D-related disorder.

B-subunits regulate holoenzyme catalytic activity, substrate specificity, and intracellular localization (6, 7). The c.1258G>A transition in *PPP2R5D* introduces a missense mutation, which replaces a negatively charged glutamic acid with a positively charged lysine (E420K) in a region predicted to regulate substrate specificity or holoenzyme catalytic activity (61) (Fig. S1*A*). We used unbiased functional approaches revealing heterozygous dominant changes in kinase/phosphatase signaling in E420K-variant cells (Fig. 4). Our data reveal increases in phosphopeptide occupancy in both HET and HOMO variant cell lines. For most peptides there was a strikingly similar increase in phosphopeptide occupancy in both heterozygous and homozygous variant cell lines as compared with WT control (Fig. 2, *G* and *H*). This indicates that the heterozygous variant introduces dominant changes in protein phosphorylation, which is consistent with the heterozygous dominant actions in patients. However, we did note instances in which the heterozygous variant had a more pronounced effect when compared with control or the homozygous variant (*e.g.*, RPS6; Fig. 5*E*), which is not entirely consistent with a dominant action of the heterozygous variant. One possibility for further studies is that the homozygous variant triggers a feedback mechanism, which due to the presence of some WT enzyme in the heterozygous cells does not reach a critical threshold needed to trigger feedback inhibition.

Our studies also reveal a role for PPP2R5D and its clinically relevant E420K variant in the regulation of AKT signaling (Fig. 7). We found that WT PPP2R5D forms a complex with AKT and PPP2AC, an observation consistent with reported co-IPs in murine adipose tissue (62) and studies in which okadaic acid or siRNA targeting the common A-subunit or C-subunit resulted in a “PP2A-dependent” dephosphorylation of AKT at Thr^308^ and Ser^473/4/2^ and reduced AKT activity. In one of the few studies that have explored the B-subunit specificity associated with AKT inactivation in dopaminergic signaling, ß-arrestin specifically recruited B55α (PPP2R2A) holoenzymes to the AKT-signaling complex, resulting in the dephosphorylation of Thr^308^ but not Ser^473/4/2^ (63). Kuo *et al.* (64) have reported that the overexpression of B55α subunit preferentially impairs phosphorylation at Thr^308^ in FL5.12 and NIH3T3 cells. It has also been shown that during AKT inactivation in insulin signaling, Cdc2-like kinase 2 phosphorylation of B56ß (PPP2R5B) regulates the assembly of a PPP2R5B-PP2A holoenzyme complex with AKT, leading to dephosphorylation of both AKT Ser^473^ and Thr^308^ (65). Finally, we have observed a strong deselection of proline-directed motifs in peptide phosphorylation that increased in the E420K variant (Figs. 3*A* and S3*A*). This is intriguing because PP2A holoenzymes containing B55-family regulatory subunits have been shown to preferentially dephosphorylate proline-directed motifs (31, 66). Together, these observations suggest that PP2A holoenzymes with unique B-subunits, like PPP2R5D, may selectively target specific phosphorylation sites on the same protein contained in a signaling complex to provide cells the ability to coordinate their growth signals *vis-à-vis* their energetic state and nutrient availability. As our studies reveal for the PPP2R5D E420K variant, aberrations in B-subunit fine-tuning of site-directed phosphatase activity can have serious ramifications on cellular growth and development. Therefore, future studies exploring differential kinase opposition by PP2A-holoenzymes should examine regulatory B-subunit composition of the complex.

Consistent with increased AKT-activation, our studies revealed increased phosphorylation of established AKT substrates (GSK3, ß-catenin, and FOXO3) and appropriate changes in downstream signaling proteins (AKT1S1, S6K, S6, EIF4B, ULK1, and CLIP1) in the E420K-variant cell lines. P-FOXO3 (Ser^253^) suppresses its ability to regulate transcription of receptor tyrosine kinases (RTKs) (67), with the inhibition of AKT inducing the expression of HER3, IR, and IGF1R. Our proteomics data show a decrease in IGF1R by 1.3-fold in HET cells (Table S2), which is consistent with the associated increase in P-AKT. Consistent with reduced IGF1R, the variant cells did not display increased P-RPS6 following IGF1 treatment as observed in WT cells. However, prolonged S6K activity has been reported to lead to feedback inhibition of IRS1 signaling (68). The variant cells have constitutively elevated P-S6, but it is premature to conclude that the attenuated IGF1–S6 phosphorylation in the variant cells is due to FOXO3 suppression of IGF1R expression. Similarly, due to complex feedback mechanisms, future studies are needed to determine the contribution of feedback inhibition in the E420K variant cells.

Cells expressing the E420K variant showed increased P-ß-catenin (Ser^552^) and increased ß-catenin protein levels, in association with decreased P-ß-catenin at Ser^33/37^ (Figs. 5, *I*–*K* and S4, *A* and *B*). Previous reports in HEK-293 cells indicate that increased GSK3 activity results in decreased ß-catenin accumulation due to GSK-mediated phosphorylation of ß-catenin at Ser^33/37^, triggering phosphorylation-mediated degradation (69). The suppression of PPP2CA has been reported to increase ß-catenin levels, due to the suppression of GSK3 phosphorylation of ß-catenin at Ser^33/37^ and the resulting decrease in ß-catenin degradation (69). Coexpression of B56 and GSK3ß has been shown to result in a substantial loss of ß-catenin levels (69), and overexpression of PPP2R5D also decreases ß-catenin levels (70). These studies are all consistent with our findings showing that the E420K variant is associated with increased AKT-activity (Ser^473/4/2^ phosphorylation) leading to increased inhibitory P-GSK3 and, in turn, to the reduction of ß-catenin Ser^33/37^-phosphorylation-dependent degradation. Of note, another study has reported that suppression of a different B-subunit (PPP2R2A) can increase P-ß-catenin (Ser^33/37^) (71). Treatment with okadaic acid has led to many conflicting observations that can be resolved if ß-catenin Ser^33/37^ is a direct substrate for B55α-holoenzymes, and B56-holoenzymes act upstream to regulate AKT and GSK3. These studies, including ours, demonstrate that distinct roles of unique B-regulatory-containing holoenzymes need to be further defined in order to fully understand the physiological complexity of cell signaling systems.

Phenotype ontology analysis of our data sets identified top genes associated with neurodevelopmental delay, with phenotypic overlap in neurobehavior observed in individuals with PPP2R5D pathogenic variants. Albeit, most were not associated with macrocephaly, a characteristic phenotype with variable penetrance in PPP2R5D-related neurodevelopmental disorder. While we are unable to draw definitive conclusions about the etiology of PPP2R5D clinical phenotypes based solely on observations made in cultured cells, our study provides evidence of convergence of common pathways for which genetic mutations have been observed in association with other neurodevelopment-disorders (72, 73, 74). Further, our cell-based data is consistent with the recent addition of PPP2R5D to the list of autism susceptibility genes (42) related to AKT3, PIK3CA, PTEN, TSC1/2, and mTOR (72, 73, 74). A key finding in our study is the demonstration of aberrant activation of the AKT-mTOR pathway, which is known to be also dysregulated in ASD (75). Increased AKT/mTOR activity is consistent with deficiencies of FMR1, TSC1/2, or PTEN found in Fragile X, tuberous sclerosis, and Cowden syndrome. Higher activity of mTOR and S6K, but lower activity of GSK3α and TSC2 have been reported in T cells from children with ASD (75). Our finding that rapamycin suppresses aberrant hyperphosphorylation of RPS6 and decreases the size of E420K variant cells beyond untreated WT should also be noted. This suggests that the development of future therapies aimed at specifically targeting PPP2R5D-dependent effects on AKT/mTOR signaling in PPP2R5D-related neurodevelopmental disorder should be mindful of potential salutary effects. The suppression of increased AKT/mTOR activity has been demonstrated to improve ASD-associated symptoms in mice deficient for PTEN and TSC1 (76, 77). If PPP2R5D-regulated signaling is conserved in mice, future studies to define E420K signaling alterations in rodent models of PPP2R5D-related neurodevelopmental disorder should further our understanding of this life-long and debilitating condition and aid the development of targeted therapeutic approaches.

## Experimental procedures

### Cell culture

HEK-293 (Clontech) cell lines were cultured in Dulbecco’s Modified Eagle’s Medium (DMEM), containing 25 mM glucose, 4 mM L-glutamine,1 mM sodium pyruvate, 10 mM MEM nonessential amino acids, 100 units/ml penicillin, 100 μg/ml streptomycin (Gibco, Life Technologies), and 10% heat-inactivated fetal bovine serum (Atlanta Biologicals, Lot# F17089) at 37 °C with 5% CO_2_ in a humidified incubator and passed when 80–90% confluent.

BE4-Gam (Addgene plasmid #100806; http://n2t.net/addgene:100806) was provided as a generous gift from Dr David Liu. Plasmids, amplified in *Escherichia coli* DH5α, were isolated using a Qiagen plasmid DNA purification kit (12123) according to the protocol of the manufacturer. All plasmids were sequenced to confirm the fidelity of the constructs. WT HEK-293 cells were seeded on 9.5 cm^2^ plates in antibiotic-free medium and transfected at ∼70% confluency using XtremeGENE9 DNA Transfection Reagent (06366511011) according to the protocol of the manufacturer. Synthetic guideRNA (crRNA:tracrRNA duplex; Invitrogen A35512) was generated as two independent oligonucleotides that were annealed. The guideRNA (targeting sequence: GCACGCUCUGCCACCUGGUG) was transfected 24 h postplasmid delivery using Lipofectamine RNAiMAX (Invitrogen 13778030). GuideRNA efficiency was first assessed using a Surveyor Mutation Detection kit following the protocols provided by the manufacturer (IDT 706020). The validated guideRNA was used in transfection, and cells were clonally isolated by plating single cells into 96-well plates using BD FACSAria II. After clonal expansion, genomic DNA from clones was isolated and PCR was performed using Q5 High-Fidelity DNA Polymerase (NEB) and oligonucleotide primers pairs that target regions flanking Exon 12 (AGAATTTTCATCCCCATGCCCTC: CAGAGATCTTGCACACAGGCATC, IDT). Sanger sequencing using ABI3730XL was employed to detect single-base mutations. Prior to further use, cell lines with the desired mutations were single-cell sorted three times to ensure each cell line represented a homogeneous population.

### Immunoprecipitations and phosphatase activity assay

Cells were plated at 1.5 × 10^6^ in 150 mm dishes and allowed to grow for 48 h. Cell lysis was achieved by the addition of ice-cold Triton X-100 Lysis buffer (50 mM Tris-HCl pH 7.4, 150 mM NaCl, 1% Triton X-100, 5 mM EDTA), containing Protease inhibitor Cocktail (ThermoScientific). Cells were mechanically sheared by gentle passage through a 28G syringe needle and centrifuged at 14,000*g* for 15 min. The supernatant was transferred to a new tube, and protein concentrations were determined using RC DC Protein Assay (Bio-Rad 5000121). TrueBlot Anti-Rabbit Ig IP Agarose Beads (Rockland Immunochemicals) were used to immunoprecipitate endogenous AKT (Cell Signaling 9272) as previously described (62), and Dynabeads Protein A (100001D) was used to immunoprecipitate endogenous PPP2CA (Millipore clone 1D6 05-421) and PPP2R5D (ab188323) according to protocols provided by the manufacturer. Immunoprecipitated samples were eluted into near-boiling 2× SDS-PAGE sample buffer and analyzed on a SDS-PAGE as described above.

For phosphatase assay, immunoprecipitated samples were eluted into 50 mM Tris HCl, 150 mM NaCl, pH 7.4. Phosphatase activity assay was performed as described previously (78). Briefly, 20 μl of IPs was dispensed in 1.33× assay buffer (0.15 M NaCl, 30 mM HEPES pH 7.0, 1 mM DTT, 0.1 mg/ml BSA, 1 mM ascorbate, 1 mM MnCl_2_) with a final assay concentration of 75 μM 6,8-difluoro-4-methylumbelliferyl phosphate (DiFMUP) (Invitrogen D6567). 6,8-difluro-4-methylumbelliferone (DiFMU) was measured every minute for 30 min with a BioTek Synergy (Ex 360 nm, Em 460 nm). Aliquots of the IPs were eluted with near-boiling 2× SDS-PAGE sample buffer and were used for western analysis to detect total levels of PPP2R5D, which was used to normalize activity measured.

### Trypsin digest of PPP2R5D peptides

PPP2R5D IPs were digested overnight with sequencing grade trypsin (0.2 μg/μl) (Promega) at 37 °C and subjected to centrifugation (10,000*g* for 5 min). The supernatant was transferred into a MS sample vial for analysis. LC-MS/MS analysis was performed with a Thermo Q-Exactive Plus mass spectrometer (Thermo Fisher Scientific). The mobile phases for the HPLC consisted of 3% ACN and 0.2% formic acid in water (solvent A) and 3% water and 0.2% formic acid in acetonitrile (solvent B). The peptide mixture was loaded onto a C18 reverse-phase column (ES800) equipped with a guard column and chromatographically separated using a liner gradient of 2%–30% solvent B over a period of 40 min at a flow rate of 300 nl/min. The peptides were subjected to electrospray ionization with positive polarity using the Thermo EASY-Spray source, with a voltage of 1.5 kV and a capillary temperature of 300 °C. Samples were analyzed in a data-dependent manner, with one MS1 scan from 400 to 2000 m/z at a resolution of 70,000, followed by the top six multiply charged ions per MS1 scan selected for MS2 scans at 17,500 resolution using HCD fragmentation. Charge states of 1, 5–8, and greater than 8 as well as those that were unassigned were excluded. The resulting files were searched using Mascot against the RefNCBInr_human protein entry databases (gi|5453954|ref|NP_006236.1| serine/threonine-protein phosphatase 2A) or Custom database (gi|5453954E420K|ref|NP_006236.1E420K| serine/threonine-protein phosphatase 2A) with *homo sapiens* taxonomy.

### Western analysis

Cells (2 × 10^5^) were plated in 60 mm dishes. For orthogonal validation of MS^3^, cells were lysed at confluent monolayers. For density analysis, low-density cells were lysed after 24 h (day 2), medium-density cells were lysed on day 3, and high-density cells (confluent monolayers) were lysed on day 4. The media was changed 24 h before lysis in independent samples of confluent monolayers at day 4 to replenish nutrients and growth factors. For growth factor analysis, 24 h after seeding, media was replaced with either serum-free or serum-containing media for 24 h before stimulation of serum-free cells with either Epidermal Growth Factor (0.5 ng/ml) (Invitrogen PHG0313), Insulin-like Growth Factor 1 (0.5 ng/ml) (Invitrogen PHG0071), or a combination of both for 30 min prior to lysing. All cell lysis was achieved by scraping cells in near-boiling 2× SDS-PAGE sample buffer (62.5 mM Tris-HCl, pH 6.8, 20% glycerol, 4% SDS, 0.0025% bromophenol blue, and 0.02% ß-mercaptoethanol) followed by mechanically shearing by gentle passage through a 28G syringe needle. Samples were centrifuged at 14,000*g* for 15 min, the supernatant was transferred, and protein concentrations were determined using RC DC Protein Assay (Bio-Rad 5000121). In total, 25 μg of each protein sample was separated by electrophoresis and proteins were then transferred onto a Trans-Blot Turbo PVDF Membrane (Biorad 10026933) at 2.5 A for 3 min using Trans-Blot Turbo Transfer System (Biorad 1704150). The membranes were blocked at room temperature for 1 h in Odyssey blocking buffer (LiCor) and then incubated over night with 1:1000 dilution of primary antibodies at 4 °C. The next day, membranes were incubated with secondary horseradish-peroxidase (HRP)-conjugated antibodies, diluted at 1:10,000 for 1 h at room temperature. Protein bands were visualized with Clarity Western ECL Substrate (Bio-Rad 1705060) using Bio-Rad ChemiDoc MP Imaging System. The same protein lysates were used to analyze phospho-proteins and corresponding total proteins or loading controls. Primary antibodies to AKT1/2 (sc8312) and P-AKT1/2/3 (Ser^473/4^) (sc7985-R) were purchased from Santa Cruz Biotechnology. Antibodies to S6 ribosomal protein (2217), P-S6 ribosomal protein (Ser^235/236^) (2211), GSK3beta (12456), P-GSK3 (Ser^9/21^) (9323), AKT (9272), FOXO3 (12829), P-FOXO3 (Ser^253^) (1312), ß-catenin (8480), P-ß-catenin (Ser^552^) (5651), and P-ß-catenin (Ser^33/37^) (2009) were purchased from Cell Signaling Technology. Antibody to PPP2R5D (ab188323) was purchased from Abcam. Antibody ß-actin (A2228) and PP2AC subunit, clone 1D6 (05-421) were purchased from Millipore Sigma. For detection of PPP2A catalytic subunit, membranes were treated with 0.2 M NaOH for 30 min at 30 °C before overnight incubation with primary PP2AC antibody. Secondary ECL donkey anti-rabbit IgG, HRP-linked whole antibodies were purchased from GE Healthcare UK Limited (NA934V) and secondary goat anti-mouse IgG peroxidase-conjugate was purchased from Millipore Sigma (A-5278).

### Rapamycin treatment

Rapamycin (MCE, HY-102019) was prepared in DMSO. 2 × 10^5^ cells were added to 60 mm dishes, allowed to grow for 24 h, and then treated with the indicated concentrations of rapamycin. In total, 10 nM concentrations were used in all FSC-H analyses. 48 h after treatment, cells were either treated with trypsin and resuspended in media to be analyzed in FACS or lysed near-boiling 2× SDS-PAGE sample buffer for western blotting analysis, as described above. FACS analysis was achieved using BD FACSAria II and Software FACSDiva Version 6.1.3. Relative FSC-H was analyzed using FlowJo.

### Quantitative proteomics and phosphoproteomics

Cell pellets from biological replicates were resuspended in of ice-cold lysis buffer [8 M urea, 25 mM Tris-HCl pH 8.6, 150 mM NaCl, containing phosphatase inhibitors (2.5 mM beta-glycerophosphate, 1 mM sodium fluoride, 1 mM sodium orthovanadate, 1 mM sodium molybdate) and protease inhibitors (1 mini-Complete EDTA-free tablet per 10 ml lysis buffer; Roche Life Sciences), and the cells were then lysed by sonication (three times for 15 s each with intermittent cooling on ice). Lysates were subjected to centrifugation (15,000*g* for 30 min at 4 °C). Supernatants were transferred to a new tube, and the protein concentration was determined using a BCA assay (Pierce/ThermoFisher Scientific), DTT was then added to the lysates to a final concentration of 5 mM to reduce disulfide bond, and DTT-lysate was incubated for 30 min at 55 °C. Lysates were then cooled to room temperate, and 15 mM iodoacetamide (room temperature) was added to achieve alkylation. After a 45-min room temperature incubation in the dark, alkylation was quenched by the addition of 500 mM DTT (1:100 dilution). After sixfold dilution with 25 mM Tris-HCl pH 8, the samples were incubated overnight at 37 °C with 1:100 (w/w) trypsin. The next day, the trypsin digest was stopped by the addition of 0.25% TFA (final v/v). Precipitated lipids were removed by centrifugation (3500*g* for 15 min at room temperature), and the peptides in the supernatant were desalted over an Oasis HLB 60 mg plate (Waters). An aliquot containing ∼200 μg of peptides was removed for proteomics analysis. The remaining peptide containing supernatant was lyophilized and stored at –80 °C until further use (*e.g.*, phosphopeptide enrichment).

Phosphopeptide enrichment was achieved using a Fe-NTA phosphopeptide enrichment kit (Thermo Fisher) according to instructions provided by the manufacture and desalted over an Oasis HLB 60 mg plate (Waters). Phosphopeptides were resuspended in 133 mM HEPES (SIGMA) pH 8.5, and TMT reagent (ThermoFisher Scientific) stored in 100% acetonitrile (ACN) (Burdick & Jackson) was added, vortexed to mix reagent and peptides. After 1 h at room temperature, an aliquot was withdrawn to check for labeling efficiency while the remaining reaction was stored at –80 °C. Once labeling efficiency was confirmed to be at least 95%, each reaction was quenched by the addition of ammonium bicarbonate to a final concentration of 50 mM for 10 min, mixed, diluted with 0.1% TFA in water, and desalted. The desalted multiplex was dried by vacuum centrifugation and separated by offline Pentafluorophenyl (PFP)-based reversed-phase HPLC fractionation performed as previously described (79).

TMT-labeled phosphopeptides samples were analyzed on an Orbitrap Lumos mass spectrometer (ThermoScientific) equipped with an Easy-nLC 1200 (ThermoScientific). Peptides were resuspended in 8% methanol/1% formic acid across a column (45 cm length, 100 μm inner diameter, ReproSil, C_18_ AQ 1.8 μm 120 Å pore) pulled in-house across a 2 h gradient from 3% acetonitrile/0.0625% formic acid to 37% acetonitrile/0.0625% formic acid. The Orbitrap Lumos was operated in data-dependent, SPS-MS3 quantification mode (80, 81) wherein an Orbitrap MS1 scan was taken (scan range = 350–1250 m/z, R = 120K, AGC target = 2.5e5, max ion injection time = 50 ms), followed by data-dependent Orbitrap MS2 scans on the most abundant precursors for 2 s. Ion selection; charge state = 2: minimum intensity 2e5, precursor selection range 650 to 1250 m/z; charge state 3: minimum intensity 3e5, precursor selection range 525–1250 m/z; charge state 4 and 5: minimum intensity 5e5). Quadrupole isolation = 1 m/z, R = 30K, AGC target = 5e4, max ion injection time = 55 ms, CID collision energy = 35%). Orbitrap MS3 scans for quantification (R = 50K, AGC target = 5e4, max ion injection time = 100 ms, HCD collision energy = 65%, scan range = 100–500 m/z, synchronous precursors selected = 5). Peak lists were generated using in-house developed software Rthur 1.0.The raw data files were searched using COMET (release version 2018.01.1) with a static mass of 229.162932 Da on peptide N-termini and lysines and 57.02146 Da on cysteines, and a variable mass of 15.99491 Da on methionines and 79.96633 Da on serines, threonines, and tyrosine against the target-decoy version of the human proteome sequence database (UniProt; downloaded 2/2013, 40,482 entries of forward and reverse protein sequences), maximum of three missed cleavages allowed, precursor ion mass tolerance 1 Da, fragment ion mass tolerance ±8 ppm, and filtered to a <1% FDR at the peptide level based on target-decoy strategy (82). Quantification of LC-MS/MS spectra was performed using in-house developed software (31). Phosphopeptide intensities were adjusted based on total TMT reporter ion intensity in each channel and log_2_ transformed. Probability of phosphorylation site localization was determined by PhosphoRS (83). *p*-Values were calculated using a two-tailed Student’s *t* test assuming unequal variance.

For proteomics analysis, peptides were labeled with TMT reagent, label checked, mixed, and off-line separated as described above. Samples were analyzed on the Orbitrap Lumos operating in data-dependent, SPS-MS3 quantification mode wherein an Orbitrap MS1 scan was taken (scan range = 350–1400 m/z, R = 120K, AGC target = 2.5e5, max ion injection time = 50 ms), followed by data-dependent ion trap MS2 scans on the most abundant precursors for 1.8 s. Ion selection; charge state = 2: minimum intensity 2e5, precursor selection range 600–1400 m/z; charge state 3–5: minimum intensity 4e5). Quadrupole isolation = 0.8 m/z, CID collision energy = 35%, CID activation time = 10 ms, activation Q = 0.25, scan range mode = m/z normal, ion trap scan rate = rapid, AGC target = 4e3, max ion injection time = 40 ms). Orbitrap MS3 scans for quantification (R = 50K, AGC target = 5e4, max ion injection time = 100 ms, HCD collision energy = 65%, scan range = 100–500 m/z, synchronous precursors selected = 10). The raw data files were searched using COMET with a static mass of 229.162932 Da on peptide N-termini and lysines and 57.02146 Da on cysteines, and a variable mass of 15.99491 Da on methionines against the target-decoy version of the human proteome sequence database (UniProt; downloaded 2/2013, 40,482 entries of forward and reverse protein sequences), maximum of three missed cleavages allowed, precursor ion mass tolerance 1 Da, fragment ion mass tolerance ±8 ppm, and filtered to a <1% FDR at the peptide level. Quantification and data analysis were carried out as described above.

### Bioinformatics analysis

Icelogos were generated using singly phosphorylated sites with a phosphorylation localization score of 0.95 or higher that significantly increased or decreased by at least twofold after protein correction in both heterozygous and homozygous variant cells (84). Kinase enrichment analysis using RegPhos was performed within Webgestalt (85) using singly phosphorylated sites with a phosphorylation localization score of 0.95 or higher that significantly increased or decreased by at least twofold after protein correction. Kinase–substrate relationships were investigated using the PhosphoSitePlus database (86). Upstream kinase prediction was carried out using NetworKIN (87). Gene Ontology (GO), and KEGG pathway analysis was performed on proteins with phosphorylation sites with significantly increased or decreased by at least twofold after protein correction in Webgestalt and visualized in Cytoscape (88). Protein–protein interactions were identified using the STRING database (89). Off-target mutations were detected using a pipeline derived from methods for detecting *de novo* mutations (90). Specifically, we used bwa-mem (91) to map the whole genome sequencing data to human reference genome version hg38, called single nucleotide variants and small insertion and deletions by GATK (92), and annotated the candidate variants by VEP (93). We define candidate off-target mutations as variants carried by the mutant but not WT cells. To exclude likely technical artifacts, we discard candidate mutations meeting any following exclusion criteria: the mutant allele is supported by fewer than six reads; allele fraction (# of reads supporting the mutant allele/# of reads covering the site) <0.3; the mutant allele is supported by reads from only one of the sequencing strands; and the genomic site is located in segmental duplication regions with score >0.95.

### Statistical analysis

GraphPad Prism version 8.2.1 was used for all statistical analyses. Image J software was used for quantification of band density in western blotting analyses. N is defined as independent biological replicates of the same clonal cell line. Passage number of the cells used in this study at the time of transfection were p3. WT control and variant cells at the time of identification of desired genetic edits were p12. All experiments shown in this study were performed at passages 12–28 (different N’s were generated between different passages to ensure no difference between cell passages were observed).

All statistical analyses are reported with standard deviation (SD). Statistical analyses were performed as unpaired *t* tests for analysis between two sample groups or Kruskal–Wallis with Dunn’s multiple comparison for multiple sample groups as indicated in figure legends. Numerical normality tests were performed to determine nonparametric statistical analysis when no assumption about the distribution of the data was possible due to small sample sizes (N ≤ 6).

## Data availability

Raw MS data for this study are available at MassIVE (MSV000084960) and PRIDE accession (PXD017562). All other data needed to evaluate the conclusions in the paper are present in the paper or the supporting information.

## Conflict of interest

The authors declare that they have no conflicts of interest with the contents of this article.

## Supporting information

Table S8

**Abbreviations**
ASD, autism-spectrum-disorder
BER, base excision repair
DMEM, Dulbecco’s modified eagle’s medium
DiFMU, 6,8-difluro-4-methylumbelliferone
DiFMUP, 6,8-difluoro-4-methylumbelliferyl phosphate
EGF, epidermal growth factor
HRP, horseradish peroxidase
IGF1, insulin-like growth factor 1
IP, immunoprecipitate
LC-MS, liquid chromatography–mass spectrometry
PAM, protospacer adjacent motif
PP2A, phosphoprotein phosphatase type 2A
TMT, tandem mass tag
WT, wild-type
