# Supplementary material for: A disorder-related variant (E420K) of a PP2A-regulatory subunit (PPP2R5D) causes constitutively active AKT-mTOR signaling and uncoordinated cell growth
Source: J Biol Chem. 2021 Jan 20;296:100313. doi: 10.1016/j.jbc.2021.100313 (PMC7952134; doi:10.1016/j.jbc.2021.100313)
Supplement: Supplementary file 1 — Figures S1 to S5 and Table S1 [file mmc1.pdf]

Supplemental Information

A disorder-relevant variant (E420K) of a PP2A-regulatory subunit (PPP2R5D) causes constitutively active AKT-mTOR signaling and uncoordinated cell growth

Cinta M. Papke[1], Kali A. Smolen[2,3], Mark R. Swingle[1], Lauren Cressey[2,3], Richard A. Heng[1], Mourad Toporsian[1], Liyong Deng[4], Jacob Hagen[4], Yufeng Shen[5], Wendy K. Chung[4,6,7], Arminja N. Kettenbach[2,3]* and Richard E. Honkanen[1]*

Content:

Figure S1:	Generation of PPP2R5D E420K Variant Cell Lines
Figure S2:	Specificity of PPP2R5D Antibody and PCA Plots
Figure S3:	Phosphomotif Analysis and Kinase Predictions
Figure S4:	Additional Immunoblotting Data
Figure S5:	AKT and PPP2A Immunoprecipitations
Table S1:	Pathogenic Variants of PPP2R5D
Table S2:	Proteomics and Phosphoproteomics data
Table S3:	Mass Spectrometry Analysis of PPP2R5D Immunoprecipitations
Table S4:	PPP-Subunit Protein Levels
Table S5:	Phosphosite Analysis and Kinase Predictions
Table S6:	Pathway Analysis KEGG and Phenotype Analysis
Table S7:	Phosphorylation sites in peptides previously linked to NDD
Table S8:	Phosphorylation sites on RPS6

Figure S1 - Generation of PPP2R5D E420K Variant Cell Lines. (**A**) Surface view illustrating the likely locations of amino acids in PPP2R5D that are mutated in the variants (pink). The PPP2CA-catalytic subunit and the PPP2R1A-scaffold are shown as grey and are derived from an existing crystal structure containing PPP2R5C and microcystin (PDB ID: 2IAE). PPP2R5D (cyan) is a homology model superimposed into the 2IAE structure. Microcystin-LR, a strong catalytic inhibitor, blocks substrate access to the active site; is shown as blue sticks. (**B**) Surveyor endonuclease mutation assay to detect generated DNA mismatches. The surveyor endonuclease recognizes and cleaves DNA mismatches due to the presence of single nucleotide polymorphisms (SNPs), which can subsequently be detected by SYBR Gold staining of the DNA after separation by size using agarose gel electrophoresis. (**C**) Workflow for the generation of triple-sorted clones. Transfected cells are allowed to grow for 72 hours for genomic editing to occur, before cells are clonally isolated through single cell sorting into individual wells of 96 well plates using an automated cell sorter. After clonal expansion, genomic DNA from ~200 clonally derived cell lines were isolated, and PCR was used to amplify genomic regions of interest. The products were analyzed by electrophoresis before Sanger sequencing to detect single base mutations. Cell lines containing the desired mutations were single cell sorted two additional times to ensure homogenous clonal population of cells.

Figure S2 – Specificity of PPP2R5D Antibody and PCA Plots. (A) Mass Spectrometry Analysis of PPP2R5D Immunoprecipitations. PPP2R5D is the main PP2A-regulatory subunit in immunoprecipitates from endogenous cells using PPP2R5D Abcam ab188323 antibody. In addition to PPP2R5D, uniquely peptides to PPP2R5E and PPP2R2A were detected, however the abundance of those were relatively minimal compared to PPP2R5D protein abundance. **(B)** Relative phosphatase activity of endogenous PPP2R5D IPs. Endogenous PPP2R5D was immunoprecipitated from WT, HET, or HOMO cells and hydrolysis of DiFMUP was measured Ex 360 nm, and at Em 460 nm. Activity was normalized to total levels of PPP2AC detected in IPs through immunoblotting shown in Fig. 1K. WT activity was normalized at 30 minutes (endpoint) to 1 and variant activity is expressed relative to WT. N=5 independent biological replicate of cells, each performed as n=2 technical replicates **(C)** Principal Component Analysis (PCA) clustering of protein-corrected phosphoproteomic samples of three independent biological samples of WT cells (WT1-3), four independent biological samples of E420K HET cells (Het1-4), and four independent biological samples of E420K HOMO cells (Homo1-4).

Figure S3 - Phosphomotif Analysis and Kinase Predictions. (**A**) Icelogos displaying 2-fold up- and downregulated amino acids surrounding the phosphorylated residue (position 0) in significantly regulated, localized, single phosphorylation sites compared to all localized, single phosphorylation sites identified in the analysis. STRING analysis of Spliceosome (**B**), Autophagy (**C**), Insulin (**D**), and mTOR (**E**) signaling networks from all changes in the protein-corrected phosphoproteome that are significantly changed in both HET and HOMO datasets.

Figure S4 – Additional Immunoblotting Data. (A) Representative immunoblot and (**B**) associated bar graphs showing the levels of P-ß-catenin (Ser$^{33/37}$) normalized to total ß-catenin in WT, E420K HET, and E420K HOMO cells (N=3 independent biological replicate of cells, mean ± SD, unpaired t test compared to WT control *P<0.05 **P<0.01). **(C)** Additional immunoblotting of density experiments shown in Figure 6M. Levels of P-S6 (Ser$^{235/236}$) and total S6, ß -actin, and Coomassie stained membrane are used as internal loading controls. Cells were assayed in 10% serum containing media at decreasing cell densities. "+" sign indicates 100% confluent cells incubated with fresh media containing 10% serum for 24 hours prior to harvest.

Figure S5 – AKT and PPP2A Immunoprecipitations. (A) Bar graphs showing the relative amounts of PPP2AC or PPP2R5D proteins co-immunoprecipitated within AKT IPs (normalized to AKT detected in IPs) from WT, E420K HET, and E420K HOMO cells. (N=3 independent biological replicate of cells; mean ± SD, ANOVA Kruskal-Wallis p=0.63 and H=1.16 or p=0.38 and H=2.2 respectively, Dunn's multiple

comparison posthoc test, NS: non-significant). **(B)** Bar graphs showing the relative amounts of AKT or PPP2R5D proteins co-immunoprecipitated within PPP2AC IPs (normalized to PPP2AC detected in IPs) from WT, E420K HET, and E420K HOMO cells. (N=3 independent biological replicate of cells; mean ± SD, ANOVA Kruskal-Wallis p=0.36 and H=2.4 or p=0.01 and H=7.7 respectively, Dunn's multiple comparison posthoc test, *P<0.05 NS: non-significant). **(C)** Bar graphs showing the levels of total AKT, total PPP2R5D, and total PPP2AC normalized to total ß-actin in WT, E420K HET, and E420K HOMO cells used as total lysate input alongside immunoprecipitations (N=4 independent biological replicate of cells, mean ± SD, ANOVA Kruskal-Wallis p=0.13 and H=4.01, p=0.41 and H=1.9, or p=0.92 and H=0.19 respectively, Dunn's multiple comparison posthoc test, NS: non-significant).

Table S1 - Pathogenic Variants of PPP2R5D

Table S1** List of all pathogenic variants identified in PPP2R5D

DNA Nucleotide Change*	Predicted Protein Change
c.157C>T	p.P53S
c.589G>A	p.E197K
c.592G>A	p.E198K
c.598G>A	p.E200K
c.602C>G	p.P201R
c.619T>A	p.W207R
c.1258G>A	p.E420K

*Reference Sequences NM_006245 .3 NP_006236 .1

*Adapted from Mirzaa G, Foss K, Nattakom M, *et al.* PPP2R5D-Related Neurodevelopmental Disorder. 2019 Jan 24. In: Adam MP, Ardinger HH, Pagon RA, *et al.*, editors. GeneReviews® [Internet]. Seattle (WA): University of Washington, Seattle; 1993-2020. Bookshelf URL: https://www.ncbi.nlm.nih.gov/books/

Table S2 - Proteomics and Phosphoproteomics data (Excel file)

Table S3 – Mass Spectrometry Analysis of PPP2R5D Immunoprecipitations (Excel file)

Table S4 - PPP-Subunit Protein Levels (Excel file)

Table S5 - Phosphosite Analysis and Kinase Predictions (Excel file)

Table S6 - Pathway Analysis KEGG and Phenotype Analysis (Excel file)

Table S7 - Phosphorylation sites in peptides previously linked to NDD (Excel file)

Table S8 - Phosphorylation sites on RPS6 (Excel file)

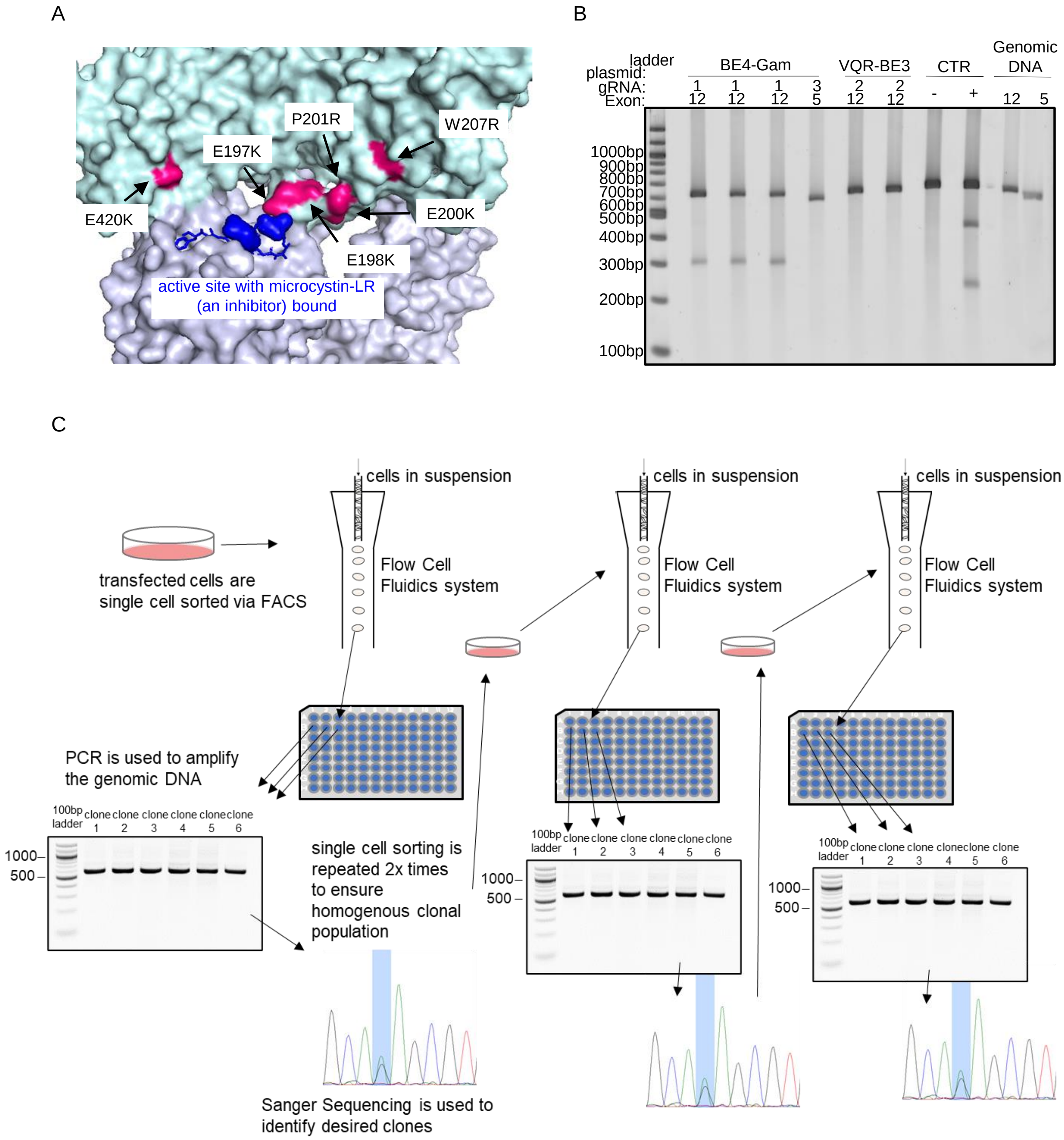
A
P201R
W207R
E197K
E420K
E200K
E198K
active site with microcystin-LR
(an inhibitor) bound
B
ladder
plasmid:
gRNA:
Exon:
BE4-Gam
VQR-BE3
CTR
Genomic DNA
1000bp
900bp
800bp
700bp
600bp
500bp
400bp
300bp
200bp
100bp
C
cells in suspension
transfected cells are single cell sorted via FACS
Flow Cell Fluidics system
PCR is used to amplify the genomic DNA
single cell sorting is repeated 2x times to ensure homogenous clonal population
Sanger Sequencing is used to identify desired clones
100bp ladder
clone 1
clone 2
clone 3
clone 4
clone 5
clone 6
1000
500

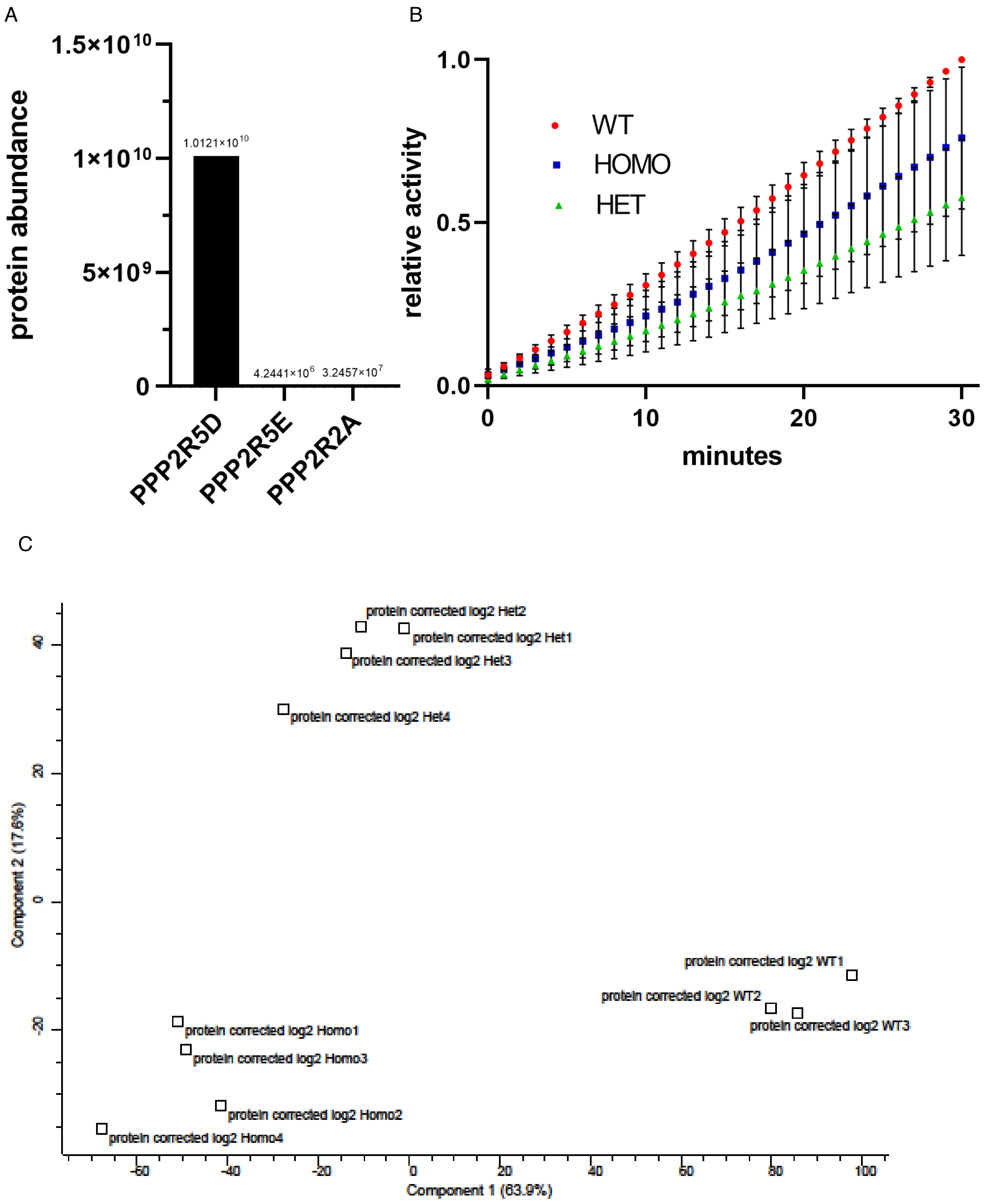
A
B
C
protein abundance
1.5×10¹⁰
1×10¹⁰
5×10⁹
0
1.0121×10¹⁰
4.2441×10⁶
3.2457×10⁷
PPP2R5D
PPP2R5E
PPP2R2A
relative activity
1.0
0.5
0.0
WT
HOMO
HET
0
10
20
30
minutes
protein corrected log2 Het2
protein corrected log2 Het1
protein corrected log2 Het3
protein corrected log2 Het4
protein corrected log2 WT1
protein corrected log2 WT2
protein corrected log2 WT3
protein corrected log2 Homo1
protein corrected log2 Homo3
protein corrected log2 Homo2
protein corrected log2 Homo4
Component 2 (17.6%)
40
20
0
-20
-60
-40
-20
0
20
40
60
80
100
Component 1 (63.9%)

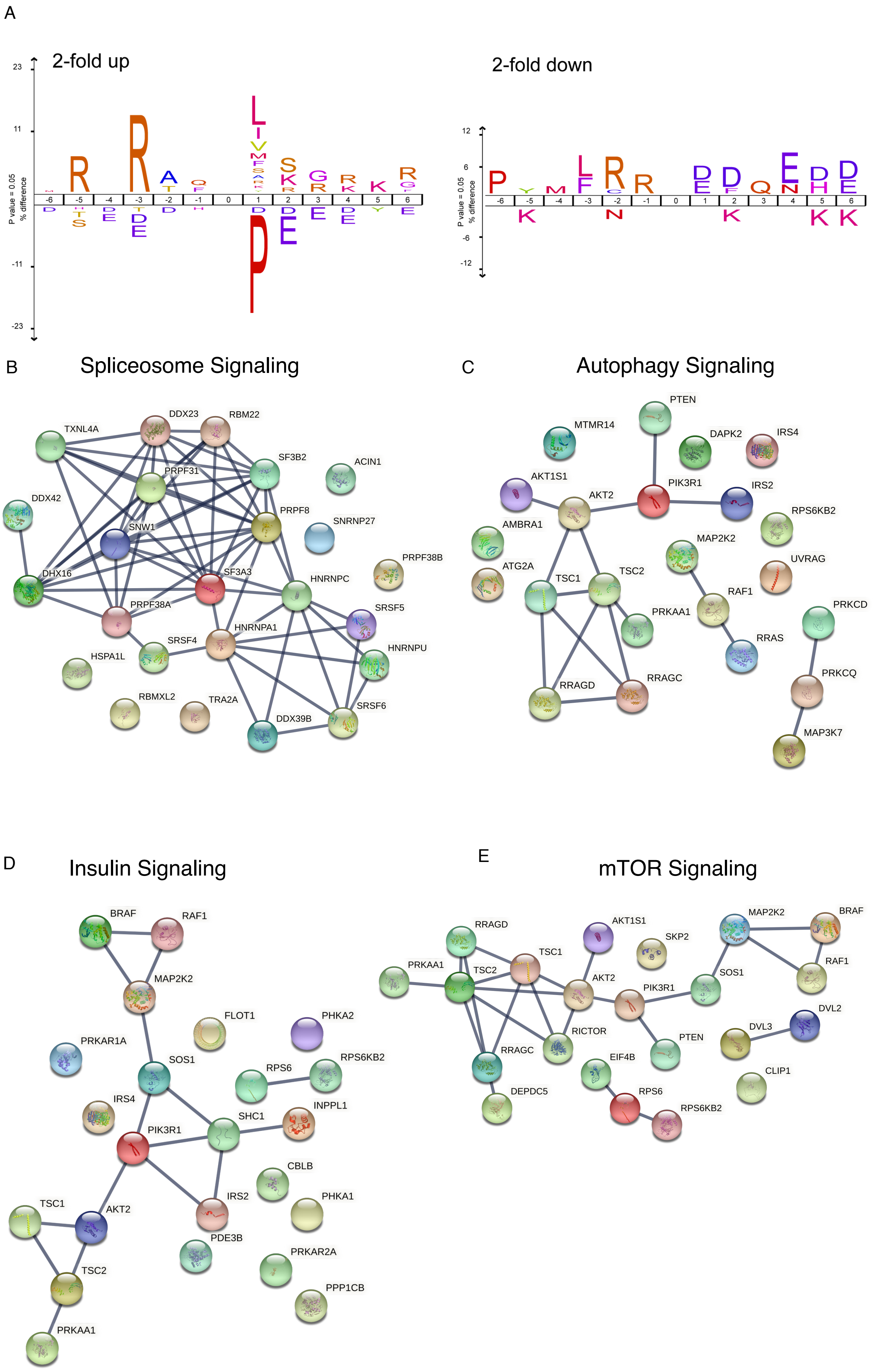
A
2-fold up
2-fold down
P value = 0.05
% difference
23
11
-11
-23
12
6
-6
-12
-6
-5
-4
-3
-2
-1
0
1
2
3
4
5
6
B
Spliceosome Signaling
DDX23
RBM22
TXNL4A
SF3B2
ACIN1
PRPF31
DDX42
PRPF8
SNRNP27
SNW1
PRPF38B
DHX16
SF3A3
HNRNPC
PRPF38A
SRSF5
HNRNPA1
SRSF4
HNRNPU
HSPA1L
RBMXL2
TRA2A
DDX39B
SRSF6
C
Autophagy Signaling
PTEN
MTMR14
DAPK2
IRS4
AKT1S1
PIK3R1
IRS2
AKT2
RPS6KB2
AMBRA1
MAP2K2
ATG2A
TSC1
TSC2
UVRAG
RAF1
PRKAA1
PRKCD
RRAS
PRKCQ
RRAGD
RRAGC
MAP3K7
D
Insulin Signaling
BRAF
RAF1
MAP2K2
FLOT1
PHKA2
PRKAR1A
SOS1
RPS6
RPS6KB2
IRS4
SHC1
INPPL1
PIK3R1
CBLB
IRS2
PHKA1
TSC1
AKT2
PDE3B
PRKAR2A
TSC2
PPP1CB
PRKAA1
E
mTOR Signaling
MAP2K2
BRAF
AKT1S1
RRAGD
SKP2
TSC1
PRKAA1
TSC2
AKT2
RAF1
PIK3R1
SOS1
DVL2
RICTOR
DVL3
PTEN
RRAGC
EIF4B
CLIP1
DEPDC5
RPS6
RPS6KB2

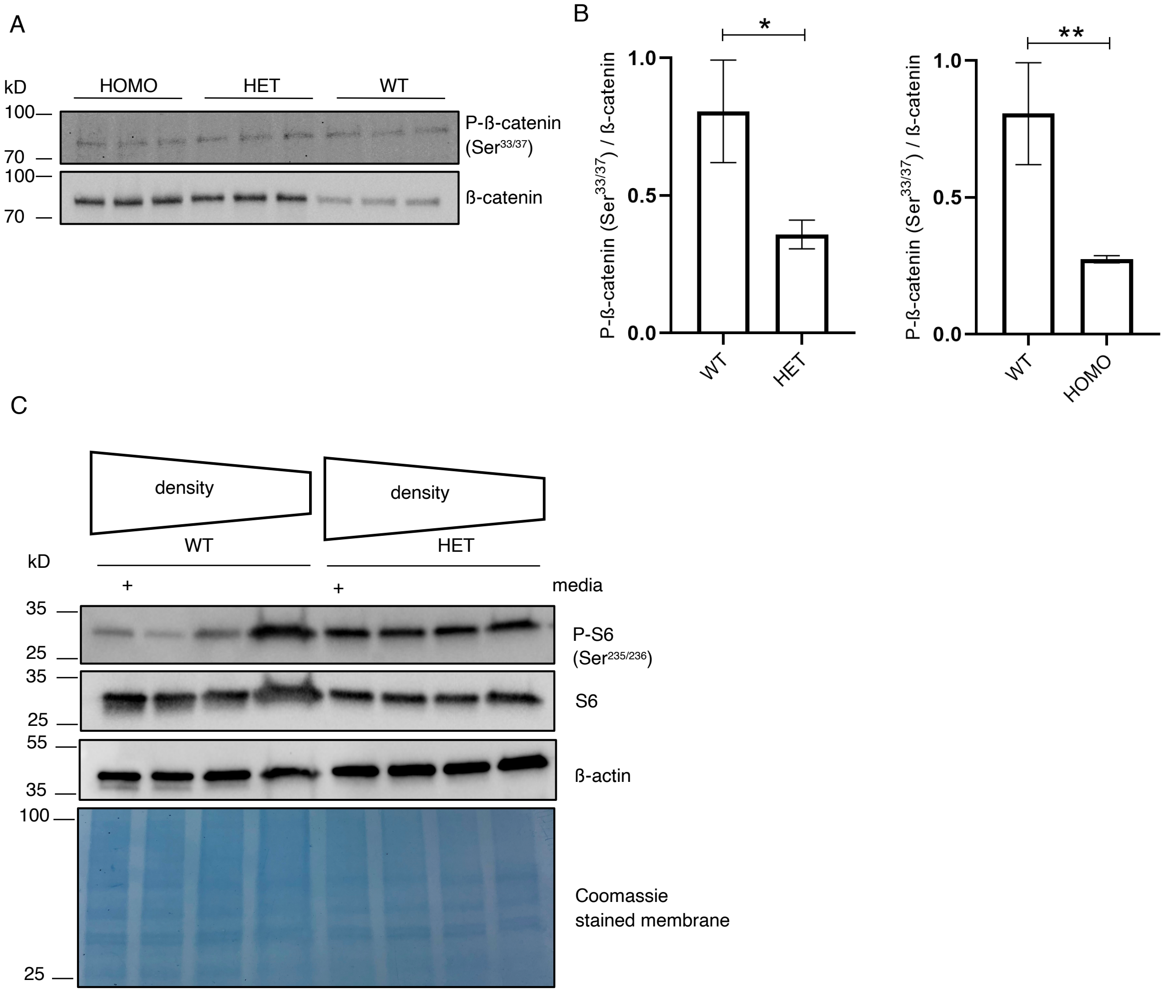
A
kD
HOMO
HET
WT
100
70
100
70
P-ß-catenin
(Ser33/37)
ß-catenin
B
*
**
P-ß-catenin (Ser33/37) / ß-catenin
1.0
0.5
0.0
WT
HET
HOMO
C
density
density
WT
HET
kD
+
+
media
35
25
35
25
55
35
100
25
P-S6
(Ser235/236)
S6
ß-actin
Coomassie
stained membrane

A

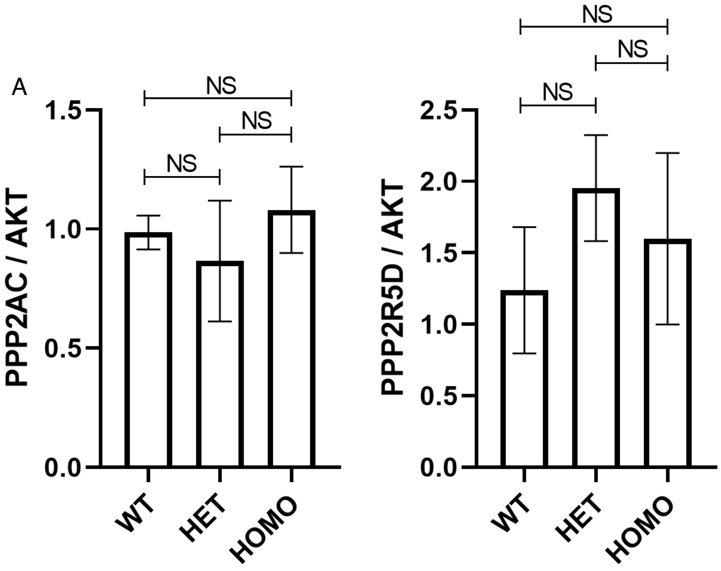
NS
NS
NS
1.5
1.0
0.5
0.0
PPP2AC / AKT
WT
HET
HOMO
NS
NS
NS
2.5
2.0
1.5
1.0
0.5
0.0
PPP2R5D / AKT
WT
HET
HOMO

B

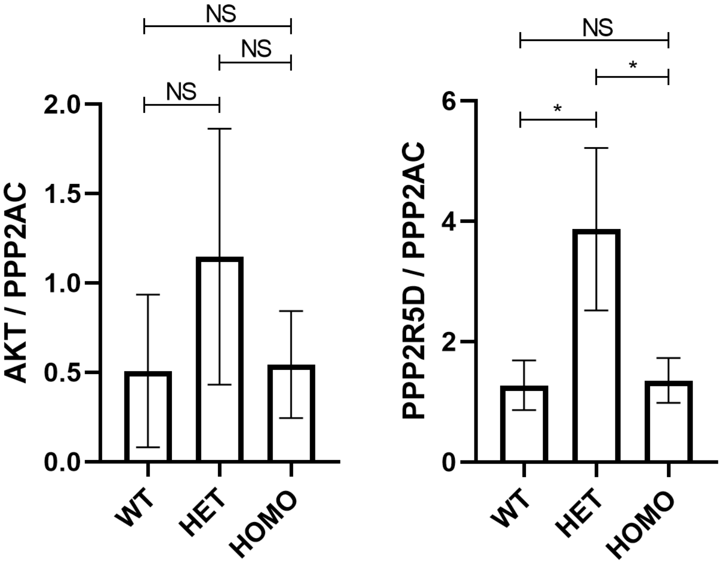
NS
NS
NS
2.0
1.5
1.0
0.5
0.0
AKT / PPP2AC
WT
HET
HOMO
NS
*
*
6
4
2
0
PPP2R5D / PPP2AC
WT
HET
HOMO

C

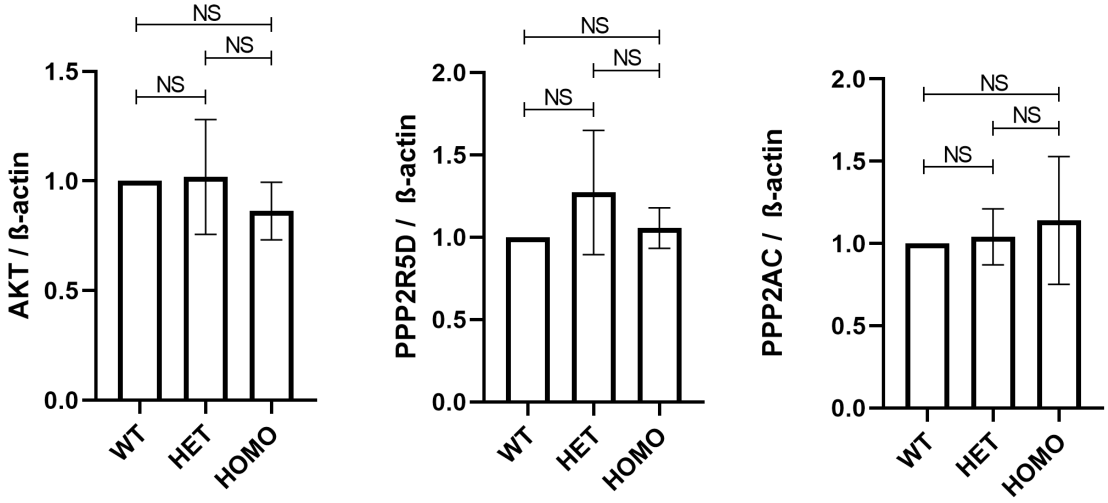
NS
NS
NS
1.5
1.0
0.5
0.0
AKT / ß-actin
WT
HET
HOMO
NS
NS
NS
2.0
1.5
1.0
0.5
0.0
PPP2R5D / ß-actin
WT
HET
HOMO
NS
NS
NS
2.0
1.5
1.0
0.5
0.0
PPP2AC / ß-actin
WT
HET
HOMO
